# Reports of Medical Cases, Selected with a View of Illustrating the Symptoms and Cure of Diseases, by a Reference to Morbid Anatomy

**Published:** 1831

**Authors:** 


### Medico-Chirurgical Review,

No. XXX.

JULY 1, to OCTOBER 1, 1831.

### I.

Reports of Medical Cases, selected with a View of Illustrating the Symptoms and Cure of Diseases, by a Reference to Morbid Anatomy. By *R. Bright*, M.D. F.R.S. &c. Lecturer on the Practice of Medicine, and one of the Physicians to Guy's Hospital. Two vols. Quarto, with 38 Plates. Longman, Rees, Orme, &c. London.

Under no circumstances can disease be treated or studied to such advantage as in hospital. In private practice experiments of uncertain issue can seldom be attempted, the wisest precepts are often violated, and the most appropriate prescriptions counteracted; cases of interest must frequently be concealed, and results, which the arrangements of a hospital would have unfailingly secured, are not rarely averted by negligence, ignorance, or obstinacy. In hospital disease can be seen in such variety of character and studied with such efficient facilities; symptoms can be traced with such unmasked perspicuity, and medicines can be administered with such watchful precision; patients can be so rigidly restricted to medical discipline, and the entire history of morbid action can be chronicled with such certainty, fulness and satisfaction, that no symptom may escape unnoticed, nor remedy remain untried. Every curative agent, which ingenuity can suggest, can be safely prescribed; and every prescription can be fairly tried, without compromising the patient's welfare, or endangering the reputation of the practitioner.

If such, then, be some of the recommendations of a hospital for the study and treatment of disease, is it not vexatious to find that medical literature owes less to the hospital physicians of this metropolis, than to any other class of the profession! It cannot be denied that, in ample scope for observation, our general hospitals are not surpassed by any similar establishments upon the Continent, and it is universally known that their medical officers are most handsomely remunerated for their services; yet it is equally certain that, in turning up the title-pages of our best works on medicine, we shall search in vain for the names of our hospital physicians. Were it not for *lecture lists*, which are periodically met with in the columns of the daily press, the very names of most of them would be unknown to the public, if not to the profession; and it is, probably, a question with many, how far the notoriety, which is acquired through the medium of pupils' fees, can enhance their literary reputation, however far it may augment their interest in the funds. It were most desirable that the governors of these noble institutions enforced it on the medical attendants, as one of the most important duties connected with their office to journal the most interesting cases of disease which come under their superintendance; and that a clinico-medical fund were appropriated from the general revenue, to defray the expence of annually supplying the faculty with such histories of disease as merited publicity. As hospitals are at present conducted in London, the public are kept in total ignorance of their internal management. The cells of the Spanish Inquisition were not sealed up from public observation with a much stricter secrecy. We neither know the number of the diseases annually admitted; their type, treatment, or duration in hospital; or the number of patients who annually recover, are relieved, uncured, or die. Hundreds of cases are yearly passing through their wards, which private practice seldom gives the private practitioner an opportunity of witnessing; and we cannot refrain from designating it an act of gross injustice to the very best interests of medicine thus to appropriate to the personal aggrandizement of a favored few most important facts, which whole centuries may not disclose in the ordinary walks of practice, and a luxuriant experience which the most unwearied private industry may be unable to collect. If our select and very privileged fraternity be too indolent to draw for themselves out of the fountain of knowledge, or too learned to profit by any further draughts from the Pierian spring; let them no longer occupy a station, which they must be satisfied they do not fill, nor cease to retard the advancement of medical science, by withholding from others what they are themselves equally unable to improve or enjoy.

With such sentiments upon this subject, it gives us unfeigned pleasure to find in Dr. Bright a very prominent exception to this general censure, and to hail his talented efforts to render it inapplicable to the hospital over which he meritoriously presides. Somewhat more than two years ago it fell to our province to bring before the notice of our readers the *first* volume of a projected series of "Medical Reports" by the present author, and the character, which was then stamped upon it, has since met with ample confirmation, not only in our own country, but upon the Continent. The *second* and *third* volumes are now before us, and in the progress of our review abundant proof will be adduced, that they are neither unworthy of their predecessor, nor of a train of subjects which has for the last twenty years been occupying the pens of our very choicest pathologists. For extent of matter and variety of detail they are, indeed, considerably superior, and the plates, with which they are enriched, are in our estimation the most perfect which British morbid anatomy can boast. From the desire of communicating a very vivid impression of the post-mortem appearances, the plates of the last volume were, probably, too richly colored; but the present pencil has laboured to avoid this common error, and whether we canvass their fidelity to Nature, or confine our criticism to the beauty of their execution, they do equal credit to the artist and the author. In the second volume are described Inflammation of the Brain and its Membranes—Acute and Chronic Hydrocephalus—Delirium Tremens—Apoplexy—Palsy—Concussion and Spina Bifida: the third contains Hysteria—Chorea—Palsy from Mercury—Neuralgia—Epilepsy—Tetanus and Hydrophobia. A catalogue of maladies much more limited and unimportant would merit extended notice; but when two ponderous Quartos, containing 700 pages, are devoted to their consideration, we despair of doing any thing like justice either to the Doctor or ourselves, in fewer than three successive articles of somewhat more than our ordinary size.

When the arachnoid is inflamed serum may be effused upon the surface, within the ventricles, or at the base of the brain;—lymph or pus may likewise be deposited in these several regions;—or adhesions may form between the arachnoid and pia mater, or pia mater and brain. The symptoms, which attend these several results of arachnitis, are somewhat different; but the lines of demarcation, by which they are separated, are often so faint and always so ill-defined, that the most experienced eye may confuse their respective limits. It is fortunate, however, that the influence of such minute diagnosis upon practice is generally unimportant. Inflammation of the brain and inflammation of its membranes, effusion upon the surface, or within the substance of the cerebral tissue, are, after all the hair-breadth definitions which have been made of them, substantially the same diseases differently denominated; and whether the serum, lymph or pus, which are effused, be deposited between the dura mater and arachnoid, or between the arachnoid and pia mater, within the ventricular cavities or upon the floor of the cranium, is more a matter of nice pathological distinction than practical arrangement calculated either to suggest or guide corresponding varieties of cure. Dr. Bright does not, therefore, attempt a division, which is unconnected with treatment, and scarcely sanctioned by symptoms. He briefly enumerates the general phenomena of cerebral inflammation, and he merely adds that—"all these symptoms suffer considerable modifications, both from the situation and acuteness of the inflammation, and from the stage of its progress and the consequent changes which the parts are undergoing." Sometimes the approach of the inflammation is insidious and its progress slow; at others its attack is as sudden as its advance is quick.

A young man (9th case), while in the enjoyment of excellent health, was suddenly seized with intense pain over the eyes, followed by severe sickness and vomiting. For the first five days he retained his intelligence, but after that period he scarcely ever spoke, and on the 15th day of the disease when he was admitted into Guy's Hospital, his face was flushed, eyes fixed and suffused, pupils rather dilated and nearly insensible, breathing sonorous, deglutition imperfect; he lay upon his back in bed, passed his dejections, which were of a dark brownish-green color, unconsciously, and he was unable to articulate. His scalp had been scarified before admission, a blister had been applied to his nape, and mercury had been exhibited till his gums were tender. Dr. Addison ordered 10 ozs. of blood to be drawn from his epigastre by cupping, a blister to be placed between the shoulders, and cold to the head; but convulsions came on, he frothed at the mouth for some hours, and died during the evening of the day on which he was admitted. *On inspection* the veins of the pia mater were found turgid with blood, the convolutions of the brain were much flattened, the medullary substance, when incised, presented more bloody points than natural, the ventricles contained three ozs. of limpid serum, the plexus choroides were unusually turgid, the vessels at the base of the brain were very full, and half an oz. of clear fluid was deposited upon it.

This man had been sadly neglected before he came into the hands of Dr. Addison; but we have some difficulty in discovering how his prospect of recovery was improved by the abstraction of blood from the pit of the stomach. His sickness and vomiting had no more to do with gastric disease, than a tender knee-joint has to do with diseased hip; and if the treatment were revulsionary, opening the saphena vein might have secured the Doctor's object more certainly. In the 68th case the suddenness of the attack is only equalled by the rapidity of the symptoms; and the extent of evil, which may exist within the head, compatible with the non-existence of all external signs, cannot be more strikingly illustrated.

A young man, 22 years of age, was treated for some febrile symptoms, in which a slight affection of the chest was the most prominent feature. After a little blood had been abstracted and a blister applied to the chest, pain in the head was for the first time complained of. It was shaved and cold water was kept constantly applied, but on the day following he became insensible, his head was drawn back upon the pillow, his right arm and leg were convulsed, his left side was semi-paralytic, his face was flushed, his skin hot, and pulse quick; and after continuing in this state for 72 hours, he died. The vessels of the dura mater and longitudinal sinus were full of dark-coloured fluid blood. The pia mater was rather vascular, and it adhered with such firmness to the brain as to be inseparable without drawing "with it a complete, thin, unbroken layer of the cineritious substance, which thus formed an even coating to the internal surface of the membrane." The substance of the brain was natural in vascularity, the ventricles contained at least three ozs. of limpid fluid, but the choroid plexus was pale, and the lining membrane did not appear unhealthy.

The intimacy and strength of the adhesions, which existed in this case between the surface of the brain and pia mater, render it tolerably evident that inflammation was working its effects upon these organs for a considerable period prior to the appearance of any cerebral symptoms; and, although such unnatural adhesions are not uncommon, it does not often happen that they are either so firm, or so extensive. The chronicity and tardiness of the 69th case will strikingly contrast with the vigor and activity of that now given.

A man, aged 56, who had for the last two years been in rather a declining state of health, but without acknowledging any pain of head or betraying any formidable symptoms of disease, was seized on the 3d of March with shivering, purging and vomiting. On the 4th he lay listless and stupid, saying that he was better and refusing to have medical advice. On the 5th he still denied having headache; but as he had no prospect of improving, a surgeon was sent for, who found him labouring under symptoms of cerebral congestion, with an oppressed pulse, dry brown tongue and unwilling to reply to any question. Next morning he became quite comatose, and 14 ounces of blood were drawn from the arm without relief. He lay on his back senseless, and nearly motionless, his left arm was bent and its fingers were somewhat convulsed, while the right lay by his side weak, but still capable of motion. His breathing was hurried and sonorous, his face flushed, skin hot, pulse slow, abdomen tumid and slightly tympanitic. The blood, which had been drawn, was covered with a thick size, and looked like badly clarified jelly. Twelve ozs. were taken from his nape by cupping, and a blister was afterwards applied; the scalp was refrigerated by an embrocation, and a purging enema was administered. In the evening he seemed rather more sensible, and gave a distinct negative to one or two questions respecting any pain he might suffer; but his eyes remained shut, his pupils were inactive, he swallowed with extreme difficulty, and after having been once more cupped on the nape to 8 ozs. he died at one o'clock on the morning of the 7th. Beneath the arachnoid a layer of puriform lymph, which had been effused into the vascular network of the pia mater, was found covering the whole superior surface of both lobes, and extended, but in smaller quantity, to the superior surface of the cerebellum, to the lateral and inferior surfaces of the brain. The arachnoid and pia mater were inseparable and easily torn, the lower surface of the latter membrane was very vascular, but the substance of the brain was quite healthy, and a quantity of fluid, very little larger than usual, lay in the ventricles.

The successful issue of the 11th case, which the Doctor considers to have been marked by symptoms of commencing effusion into the ventricles, must be ascribed to repeated blistering, and the continued exhibition of grain doses of calomel. A man, aged 34, who had occasionally during four years suffered from pain in the head and limbs, for a month before his admission into hospital was afflicted with intense headache, for which he had been cupped and bled with partial relief. When admitted his vision was so far impaired as to be unable to distinguish the figures on a watch, he complained of numbness of the left arm and thigh, his countenance was dull and vacant, the pain, of which he complained in his forehead, was principally confined to the right side, his articulation was slow and his pulse oppressed. One grain of calomel was at first given every eight, and afterwards every four hours until his gums became tender, when it was again exhibited thrice daily, and the nape was kept constantly blistered. The pulse gradually rose from 62 to 72, 80, and at last 100; his eyes became less dull, he walked with a firmer step, his headache ceased, and in about six weeks he left the hospital without complaint.

The cases, which we have now extracted, are well calculated to illustrate some important peculiarities in the manner, in which ordinary arachnitis makes its approach and proceeds to its termination in ordinary constitutions. But where the nervous system has been impaired by debauchery, and where there is more irritability than phlogistic diathesis, inflammation of this membrane is marked by symptoms and requires treatment which have not yet been mentioned. *Delirium tremens*, we need scarcely add, is the affection to which in this observation we allude.

A robust woman, of 30 years of age, whose habits were intemperate, had been seized with a fit of delirium for which she was bled, blistered and purged; but as she had afterwards been occasionally delirious, she was placed under the Doctor's care. When he saw her she spoke collectedly, but her manner was very hurried, her actions were quick, her pulse was 108, sharp, she was thin, her tongue was furred, dry and rough. Fourteen ozs. of blood were withdrawn, a pill containing two grs. of calomel and four of hyosciamus was given every four hours, and a little oil was ordered for her bowels. The blood was inflamed, but the operation did not produce any good effect, for towards evening delirium came on which required restraint, and her stools were passed unconsciously. Venesection was repeated to the same extent, the quantity of calomel contained in the pill was doubled, the head was shaved, and cold was applied. Temporary relief followed the bleeding, but soon after she became delirious and continued so throughout the night. A quarter of a grain of tartar emetic was added to each pill, and as the bowels were confined, half an ounce of oil was given. During the next four days no change occurred under this plan of treatment, after which her mouth became sore, delirium subsided, and the pills were omitted. With the exception of occasional pain in the head, for which a few leeches were applied to the temples, and some tartar emetic ointment was rubbed on the nape, her cerebral symptoms gradually subsided, and after six weeks attendance she was dismissed cured.

In this case there was obviously considerable inflammatory action, and from the appearance of the blood, the violence of the symptoms, and the state of the pulse, some might not have restrained the lancet to a second application ; but from the intemperate character of the patient, the extreme irritability of her constitution, and the evident increase of delirium, which followed each depletion, we have little doubt that while the lancet may have prepared the habit for the more effectual and rapid operation of mercury, it is to this medicine we must especially look for the complete and permanent removal of the disease. In the sixth case, which is from the pen of Dr. B. Babington, the symptoms and treatment are extremely similar. Bleeding had been employed at first with temporary, but afterwards equivocal relief, and suspecting that the lancet had done no good, Dr. Babington adopted the soothing plan. Opium in powder, tincture and pill was given in liberal doses, the delirium, watchfulness, and irritability subsided, and in less than a week he was in a state of perfect convalescence. In the fourth case the patient was bled in the recumbent posture to 24 ozs. when he became faint, and six leeches were applied to his temples, which had the effect of rendering him less violent, but did not abate his delirium ; in the fifth case local bleeding, both by leeches and cupping-glasses, was tried with only temporary relief ; in the seventh case the leeches, which had been applied to the temples, materially aggravated the symptoms ; and in the thirtieth case depletion was tried on rather a large scale with very doubtful effect ;—while every instance, in which soothing treatment was early and steadily pursued, either rapidly recovered, or showed in the subsequent alleviation of it symptoms how much more congenial was such a plan to the nature of the action upon which the disease depended. In the 67th case the patient was found in a state of violent agitation requiring restraint, his pulse was frequent, manner quick, and his eyes were constantly in motion. Cold was applied to the head, five grains of hyosciamus and two of calomel were ordered every four hours, and his bowels were freely excited by opening medicines. On the fifth day his mouth was tender, his mind gradually regained its tranquillity, and he rapidly got well. This man had been much addicted to drinking for some days before his attack; he had occasionally felt, while on the streets, momentary aberrations of mind, and when conveyed to the hospital he fancied that he had been accused of some crime, was going to prison, and would be executed.

It is now, we believe, tolerably clearly ascertained that in most cases of delirium tremens, the constitution is unable to withstand much active treatment, and that, although the membranes of the brain are generally in an evident state of inflammation, the local disease is so modified by the enervated condition of the general system, that if depletion be at all admissible, it must be employed early, cautiously, and in conjunction with such medicines, as are more caculated to diminish action by obtunding excitement, than by depressing strength.

"It cannot certainly be laid down as a rule that in such cases bleeding must never be had recourse to; but that it should be adopted with much caution cannot be doubted, and it should be immediately followed by the administration of opiates combined with calomel. The very early use of tonics, and even stimulants, will in many cases be advisable; and improvement in the diet, more particularly the substitution of some solid food, together with a limited quantity of malt liquor, instead of slops, will be beneficial; and in all cases the strictest attention must be paid to the removal of every source of mental disturbance and excitation; and with this view, all restraint, except such as is absolutely necessary for the safety of the patient and his attendants, should be avoided. The head should be constantly cooled by evaporating washes, and the atmosphere should be kept as cool and as pure as possible. The calomel, in conjunction with more or less opium or hyosciamus according to the degree of irritability, should be continued till the symptoms have completely subsided, or the mouth is affected." 26.

The membranes of the brain, like every other part in the animal frame, may suffer, not directly, but *by sympathy with diseased organs*, situated at a distance from them. Thus, irritation of the brain may be induced by hepatic, gastric, or intestinal disorder; this irritation may pass into more active derangement, if the operation of the cause continue; inflammation may ultimately be established, and the original disease may become so masked by the cerebral affection, or so secondary in importance, as at length to escape all notice. In Dr. Bright's first volume of Reports several cases of this description were detailed while upon affections of the intestinal lining in fever, and a few more are here added to complete the series.

A young lady (Case 31) contracted a slight cold while in London on a visit from the country, for which five grains of calomel were taken. The next day, however, brought no alleviation of the symptoms, cerebral excitement came on, she was regarded as labouring under an attack of phrenitis, and was treated accordingly; but she sunk on the fourth day of the disease without betraying any unusual tenderness in the abdomen. On opening the head not the least trace of disease could be detected; but the lower part of the mucous lining of the ileum was studded with diseased glands in various stages of enlargement; the valve of the colon was covered with a layer of coagulable lymph, and the colon itself was similarly affected.

On two important accounts this history is valuable. It strongly shews how far intestinal disease may proceed without producing abdominal tenderness; and how violent inflammatory cerebral symptoms may appear, without leaving behind them, after death, upon either the membranes or substance of the brain, the slightest vestige of disease. In the 33d case, what is considered the secondary affection of the brain was much more serious; but to us it is very questionable whether, in this instance, the cerebral disorder were not coeval with the intestinal disease. In fever, nothing is more common than to see the head and the abdomen suffering simultaneously from the commencement, and proceeding in intensity with equal step throughout the entire course of the disorder. The girl, whose case is here given, had been labouring under fever for a week prior to admission, and no account is given of the symptoms worn by her complaint during that period. When first seen by the Doctor, she was delirious, very restless, constantly tossing her arms about her face, was covered with perspiration, her tongue was dry and brown, her pulse was 120, she neither complained of head-ache, nor abdominal tenderness, had much subsultus tendinum and her pupils were dilated. One grain of mercury was given every six hours, cold was applied to her head, a blister to her nape, and she was ordered a purging enema. On the next day, her pulse was 145, her respirations were 60, a tendency to ecchymosis appeared in various parts, she was more comatose, her hands were cold, and she died on the following morning. The vessels of the brain were generally turgid, its substance was unusually vascular, and some serum was effused beneath the arachnoid and upon the base. The lining membrane of the stomach was vascular, and in some parts slightly ecchymosed; the mucous surface of the small intestines was also vascular, the mucous glands in the duodenum were considerably enlarged; about a foot of the lower part of the ileum contained patches of thickened and elevated aggregate glands, some of which were passing into ulceration; the cæcum was very dark and vascular; the glands of the mesentery were purple and enlarged.

The next case is from the pen of Dr. Dill: but as many of its features are of a very peculiar character, it shall be noticed on another opportunity. The 37th history strikingly illustrates how violently the brain may sympathize in an irritable habit from a very trifling injury received by one of the lower extremities.

A robust seaman, aged 25, who had once been much addicted to intemperance, received a contusion on the knee, which swelled and became painful, but was gradually improving, when he was suddenly seized, during night, with head-ache, stiffness in the neck, pain in the back and loins, vomiting and purging. Two days afterwards, both legs became swollen and painful, symptoms of general fever appeared, succeeded by delirium, and, when admitted into hospital, there was a small ill-looking ulcer on the left patella, and the back part of the leg was swollen, tense, red, and acutely sensible; but the knee-joint was unaffected. The right leg was still more tense and painful. Powders, composed of tartar-emetic, opium, and calomel were given every six hours, with a saline mixture; cold was applied to the head, and a warm spirit-lotion to the legs. The general symptoms unabating, and the right leg and the foot being threatened with gangrene, Mr. Key made a free incision, of considerable extent, upon both the inner and outer sides of the leg, which gave issue to a small quantity of bloody serum.

The cellular tissue was not much infiltrated, and the muscles appeared healthy. A poultice was then put over the limb, porter and other means of support were ordered, five grains of the extract of hyosciamus were given every four hours, a sinapism was placed on the epigastre, and the bowels were urged with opening medicine. The delirium and every other symptom continued unabated, and stimulants were given in large quantity, but he gradually sunk during the evening of the next day. No disease could be discovered in the contents of the cranium, if we except as such an unctuousness on the surface of the arachnoid; the heart was very much contracted, entirely closing up its left ventricle; the surface of the left lung was slightly ecchymosed, and a quart of brown-coloured serum occupied the left pleural sac; the spleen was soft, but the other abdominal viscera were quite healthy. The right leg was livid, the cuticle about the upper part was raised, the cellular tissue surrounding the muscles was infiltrated, inflamed, and in some parts sloughy, the soleus was soft, and its surface was stained of a dark colour. The left leg was similarly diseased, but not to the same extent. The veins of both limbs were quite healthy.

In the 42d case, a gentleman who had lived for some time between the Tropics was the subject of disease. When seen by the Doctor, he exhibited marked symptoms of cerebral excitement. His mental faculties were unusually active, his eye was red and glistening, and he complained of headache. The colour of his face was icteroid. On pressing the region of the liver, he immediately evinced signs of pain. After being cupped from the nape, bled from the arm, and taken several calomel purges, he was more mild; but it was only after the re-establishment of the biliary secretion, by cupping over the liver, and affecting the mouth with mercury, that his natural complexion returned, and his cerebral symptoms disappeared.

There can be no doubt of the existence of hepatic disease in this instance, and as little of the relationship between that disease and the cerebral affection. The general bleeding and the blood, which had been drawn from the nape, no doubt checked the progress of the secondary symptoms; but until the exciting cause was effectually removed, by restoring the functions of the liver to their natural state, the brain continued to labour under the effects of sympathy. When the scalp, or skull is injured by external violence, the inflammatory action thus awakened not unfrequently extends to the membranes of the brain, and the same consequence is often witnessed in erysipelas of the face and head.

It has long been a question with medical men, whether those, who are labouring under *erysipelatous disease*, should be considered capable of imparting that disorder to the uninfected, and the profession have regularly marshalled themselves into two parties upon this controverted point. If the reader will refer to our review of Mr. Lawrence's paper on the treatment of erysipelas which attracted considerable notice about two years ago, they will there meet with some very interesting materials in illustration of this question. Several cases and some important authorities were then brought forward in proof of the contagiousness of this disease. Some indisputable evidence to the same effect has occurred to us since, and we are now as well assured that some forms at least of erysipelas are endowed with as communicable a property as typhus fever, small-pox, or measles. It is gratifying to find an observer of Dr. Bright's opportunities and talents throwing the weight of his authority into the scale of the contagionists. The following interesting cases will be read with attention.

The wife of the patient (49th case) whose case is given in the 48th history, which will be immediately detailed, and who had assiduously attended him during his illness, was seized with symptoms of erysipelas just as his attack was on the wane. Punctures were employed in three or four instances, but with little success; the inflammation spread from the face over the upper part of the body, and terminated at last in abscess on the right side of the neck.

(50th Case.) One of the nurses, who had charge of several very severe cases of erysipelas, was seized with febrile symptoms, which were succeeded by erysipelas of the nose and right cheek. A great number of punctures "amounting to some thousands" were made over the inflamed surface, and they were sponged with warm water. These punctures were repeated, at least, twice every day, little or no vesication took place, and although her fever ran high, she betrayed a strong tendency to sleep, and much despondency, no medicines, but the simplest salines and gentle purgatives, were required, in addition to the local treatment, to complete the cure.

A stout muscular man (Case 44th), aged 37, who had been very intemperate, was attacked by erysipelas of the face, just as his wife was convalescing from a similar affection. After two days he was delirious, opium and wine were ordered in large quantities, and blood was taken from the arm; but, from the supervention of syncope, to a small amount. When first visited by the author he was prostrate on his back, constantly talking and occasionally laughing. The left side of the face was covered with erysipelas of a dark colour; the pulse was 120, weak; tongue dry; and when closely pressed he acknowledged some headache. His head was shaved and kept cold, sinapisms were applied to the feet, blisters to the nape, opium and calomel were freely given, his bowels were preserved in an active state; and means were had recourse to for the support of his strength. The disease, however, extended over the scalp, the breathing became laborious, the pulse more frequent, the evacuations were passed involuntarily, the nates excoriated, delirium gradually subsided into coma, and he died four days afterwards. A considerable quantity of serum was infiltrated into the integuments of the scalp, the arachnoid was thickened and opaque in several parts, and contained beneath a considerable quantity of serous fluid. The large veins of the pia mater were turgid, the substance of the brain was pale and flabby, a small quantity of secretion lay in the ventricles, and much at the base of the brain and in the spinal sheath.

Dr. B. strongly reprobates, in the treatment of erysipelas of the head, the indiscriminate employment of stimuli, as peculiarly calculated to do irreperable evil. As a general rule he would abstain from all such measures, until the patient's strength imperatively called for them, and in the early stages he would bleed, generally from the arm and locally from the nape, purge mildly, and produce gentle diaphoresis. He very justly lays much stress upon the local treatment.

"Blisters to the nape of the neck, and that particular mode of scarification which has been recommended by Dr. Dobson, of Greenwich Hospital, which, as far as I have myself had an opportunity of watching its effects, I consider one of the greatest improvements in modern medicine. This consists in making fine punctures, in number amounting to several hundreds or even thousands, with the point of a lancet over the whole inflamed part; then fomenting with warm water in a sponge, to encourage the bleeding; and repeating this operation two or three times in the twenty-four hours, if the parts look red or tense. If done early, it shortens the disease; but at all events it relieves the vessels in a manner which nothing else in my experience has effected." 97.

We have had very frequent opportunities of verifying this favourable character of acupuncturation in erysipelas. In many instances we have arrested the further progress of the disease by making numerous fine punctures over the inflamed part, and we have found it of great importance to puncture the circumference of the diseased surface more abundantly than the centre, or interior. If small punctures be very liberally made around the boundary line of the disease, as soon as it makes its appearance on the face, and if the other antiphlogistic measures now specified be judiciously employed according to the strength and habits of the patient, we shall in many instances circumscribe the evil within its original limits, as effectually as they do in France by applying blisters along the inflamed margin; and with much less pain. If, however, this treatment be neglected until the disease have made considerable progress upon the skin, until vesication have appeared, and the brain begins to sympathize; the good, which it effects at this eleventh hour, is partial, and it seldom, so far as we have seen, has curtailed the further march of the inflammation. Punctures may be made in parts of the body and in constitutions in which lengthened incisions would be wholly inadmissible; but we have found them of greatest value in erysipelas of the face, and where the strength had been seriously reduced by previous disease. We prefer the point of a narrow-shouldered lancet to any form of needle which we have yet seen; and while the depth of the puncture should be regulated by the depth of the disease, we have generally found it useful to empty as many of the superficial vessels as possible. The two succeeding cases will show, not only the value of this remedy, but also the degree to which the period of its application influences its effects.

A young woman (case 45th), who had just recovered from an attack of pneumonia, was seized with erysipelas of the face, beginning on the nose, spreading over the whole face and scalp, and at last invading the lining membrane of the nostrils and fauces. The scalp, forehead and face were punctured twice, and fomented during the first day. The relief produced was great, the incipient delirium, which had appeared, was checked, and the inflammation subsided. Puncturation was repeated the following day with equally good effect, but when the external disease was thus so far reduced delirium returned, for which a blister to the nape was considered necessary. This was removed, and the cure was completed by slight tonics.

(Case 48th.) A middle aged man was admitted into hospital labouring under erysipelas, and almost in a state of coma. The right side of his face and nose, part of the left cheek and throat, back of his neck and shoulders were swollen and red with erysipelatous inflammation. His manner was hurried, pulse 140, respirations 30, and his bowels purged. His head being shaved punctures were freely and repeatedly made over the forehead, nose, cheeks and chin, the bleeding was encouraged by warm sponges, and he took some saline mixture. On the next day the inflammation was obviously diminished, but still considerable, the eyes were closed up and the whole scalp was swollen. The face, scalp and ears were again punctured, and a small artery in the scalp, which the lancet had opened, poured out at least half a pint of blood, and ultimately required to be divided. A purging enema and a febrifuge mixture were given. During the evening the punctures were again repeated, next day the swelling both of the face and scalp was greatly relieved, and four days afterwards the face had desquamated, the tumefaction has subsided, and the erysipelas, which had travelled slowly down the back, had assumed the gentlest character.

In the 51st Case a young girl, labouring under syphilis, was attacked by erysipelas of the face. The head was shaved, punctures were made twice daily, a saline mixture was employed, and in a short time she got well. In the 52d Case a paralytic patient became the subject of this disease, and punctures were had recourse to; but although the right cheek alone was first invaded, the whole face and scalp were ultimately involved. So early as the second day of the disease, the strength required support, first in the form of beef-tea and infusion of serpentaria, and afterwards by means of wine and ammonia. Very little vesication occurred, the face desquamated freely, and complete recovery followed. A man labouring under disease of the stomach was the subject of the 53d case. On the 24th of November erysipelas appeared upon his face, he became delirious, his hands tremulous, and his tongue brown. The affected part was liberally punctured four times, and salines were freely given. In less than a fortnight this patient was convalescent. Before this attack of erysipelas his stools had been mixed with blood, and untinged with bile; but for several weeks after his recovery, bile was secreted in sufficient quantity, and his stools resumed their natural color. In the 54th and last case a man, who had been for years subject to severe attacks of ague, which had evidently diseased his spleen and much reduced his general strength, and who had in February passed through a dangerous attack of small-pox, was seized in March with erysipelas of the right leg, which was treated by punctures. To this succeeded severe erysipelatous inflammation of the face, which was overcome by the same means, and he left the hospital on the fifth of May with no other disease than the enlargement of his spleen. No one can attentively examine the symptoms, treatment and results of these cases, without attaching considerable importance to puncturation in the accomplishment of their cure. In every case, in which it was employed early in the disease, the violence of the general symptoms evidently abated, and the progress of the local affection was obviously retarded, when not arrested. And in no instance here detailed, or that we have yet seen, has this practice been followed by any bad effects.

"I do not remember to have seen a fatal case where the punctures were used early and persisted in strictly. At the same time, I should be very sorry to be considered as advocating the infallibility of this or any other remedy: for no doubt cases occur which will prove fatal under any treatment; and as there is sometimes an insuperable difficulty in persuading the patient to submit to the use of this particular mode of treatment, it is satisfactory to know, that the majority of the cases of Erysipelas of the face and head do well whether punctured or not, provided the treatment be tolerably judicious in other respects:—still, however, from a comparison of the cases which came within my knowledge before I had adopted this treatment, and those which have occurred since, I am strongly persuaded of its utility. It affords local ease, and checks the severity of the cerebral and general symptoms; and if early employed, it prevents in a great degree the vesication, and, I think, diminishes the chance of suppuration in the soft and cellular structures, particularly about the eyes, where the formation of pus is very apt to take place. Provided the punctures are very minute, and not lengthened into small incisions, I have never seen them leave any permanent marks, even on the smooth skin of the forehead; nor am I aware of any circumstances of inconvenience or danger which can be regarded as affording a solid objection to this practice. As, however, there is a possibility that the punctures may sometimes remain visible, it will be right to take particular care in puncturing the parts most exposed to view. 105.

Inflammation of the lining membrane of the nose and frontal cells has been found in some instances to extend to the interior of the scull, and occasion delirium, coma, and the other usual consequences of severe phrenitis. The late Mr. John Pearson attended a child, who had been first attacked with erysipelas of the nose, and was subsequently seized with delirium and coma, under which it died. In a case recently witnessed by Mr. Toulmin of Hackney, after great discharge from the nose the patient died from the supervention of severe cerebral symptoms; and inspection after death discovered pus in the frontal sinuses, together with a cyst of muco-purulent fluid in one of the anterior lobes of the brain. Instances of chronic inflammation of the mucous membrane of the nose are not unfrequently seen to destroy the cartilages and bones of the head, and ultimately to affect the brain itself. At the close of catarrhal and continued fever, inflammation of the lining of the external passage to the ear and of the cells of the temporal bone is, however, still more common. Where the leading symptoms have been chiefly cerebral, and where the cerebral inflammation has not been completely subdued, ear-ache is a very common secondary affection in the close of fever. Throbbing pains are at first experienced around the region of the external ear, these are succeeded by a discharge of a fluid, which is at first serous, but afterwards becomes puriform, and the pain decreases on the increase of the discharge.

In general this affection has been found gradually to subside without any peculiar treatment. In some deafness, vertigo, and a distressing sense of noise in the head have complicated the other symptoms, and retarded recovery. And in a few delirium, convulsions, paralysis, and coma announce the extent to which the brain has suffered, and mark the closing periods of life. In such severe cases the bones of the ear become carious and are discharged, the membranes of the brain inflame and ulcerate, and the cerebral substance is disorganized. Scrophulous tubercles and fungoid tumors are occasionally discovered, and it is not unusual to find pus collected under the pericranium behind the ear, with the dura mater internally detached, and the temporal bone denuded. The Doctor has witnessed cases where, after discharge from the meatus externus, an abscess has opened behind the ear, bone has exfoliated, and recovery has been obtained with the sacrifice of the sense of hearing on that side.

A young girl (Case 55th) was labouring on the 3d of February under low fever, gastric derangement, and indications of a chlorotic habit. On the 14th great pain of the right ear was complained of, three days after a copious discharge of serous fluid appeared, which on the 19th assumed a puriform character. The pain gradually subsided, and with the exception of slight deafness for a few days all the symptoms disappeared. (Case 57th.) A little boy became the subject of malignant scarlatina. His head-ache was considerable, and the cerebral disturbance was so serious as to demand leeches to the temples. A serous discharge afterwards commenced from the ear, which on the day following became purulent. When first seen by the Doctor the whole auditory canal of both ears was lined with little vesicles, like the glands on the ice-plant, and the discharge was acrid and profuse. Mild astringents were employed, and as the child recovered from its fever the secretion declined.

A woman, (Case 58th,) aged 27, ill with scarlatina, complained much at first of pain in the upper part of the head, which gradually descended towards the ears. Five days after this time the left ear became painful and began to discharge; for which blisters were placed behind the ears. Considerable fever, with rigors, head and ear-ache ensued; pulse 125; skin hot. Saline and diaphoretic medicines were ordered, and the nape was blistered. Four days afterwards peculiar stupidity was complained of. This feeling increased to a sense of tipsiness, the stomach became disordered, but the discharge from the ear ceased. She had no head-ache nor delirium, and by keeping a blister between the shoulders for some time open, by relaxing the bowels, and giving gentle tonics, she was dismissed cured.

Such cases as these are slight and almost independent of medical treatment; but those which follow are more formidable and less easily cured.

A sailor, (60th,) aged 30, was admitted into hospital on the 23d of November with symptoms of fever, for which he was ordered a diaphoretic julep, a blister to the chest, and some castor oil. On the 2d of December he was much purged, his tongue was dry and brown, his voice got hoarse, and his pulse was 120. Compound chalk-powder and the hyd. c. creta were given to appease his bowels, an opiate linctus was ordered for his cough, and the blister to his chest was repeated. On the 5th there were urgent symptoms of fever. A calomel, opium, and tartar-emetic powder was given at night, ipecacuanha and conium three times daily, and the febrifuge mixture. By the commencement of January he was convalescent, and on the 17th a purulent discharge from the left ear for the first time appeared. Blisters behind the ears and upon the nape, kept open with savine ointment, were had recourse to; but on the 22d, a puffy swelling showed itself behind the left ear with increased head-ache, for which ten ounces of blood were drawn from the neck by cupping. On the 6th of March this swelling was opened, and discharged five or six ounces of pus. On the 25th it had again filled and was again emptied. From that time forward every formidable symptom declined, and he was dismissed cured on the 1st of May. Dr. B. believes that the dura mater must have suffered in this case, but the happy issue of the disease does not permit us to imagine, that the injury which it may have suffered was considerable. The 63d case is worthy of notice, being probably an example of scrophulous tubercle excited in the brain of a strumous patient by diseased ear.

A little girl, four years old, had for some weeks been afflicted with pain in the left ear, attended by a remittent discharge of puriform fluid, often tinged with blood. Hearing on that side was much impaired, the sleep was disturbed, and she had all the signs of low irritative fever. The pulse was 110, quick and wiry, the skin hot, she complained of head-ache, and was occasionally convulsed. There was a direct ulcerated communication between the meatus and mastoid cells. Leeches were thrice applied to the ear, with the effect of diminishing the pain; laxative and sudorific medicines, with small doses of calomel, banished the febrile symptoms; a bread and water poultice was applied every four hours to the diseased part; infusion of cascarilla with muriatic acid were given to improve the stomach, and she had every appearance of doing well, when she suddenly grew worse, was frequently convulsed, and died. The integuments behind the ear being divided, a ball of dead bone was easily detached from a carious cavity, formed partly out of the mastoid cells and auditory canal. When the probe was introduced into this cavity, it touched the dura mater and easily passed into the eustachian tube and cavities of the labyrinth. The left lateral and petrosal sinuses were filled with firm coagula. There was little serum in the ventricles, and none between the membranes of the brain. Close to the left ventricle, imbedded in the cerebral texture, lay a tubercle as large as a walnut, which was removed almost without dissection. The fore part of the cerebellum was stained with a purple hue, and in it was found another tubercle in the first stage of suppuration. Two small ulcerated apertures were discovered in that portion of the dura mater, which lines the temporal bones, and the outer surfaces of this membrane were coated with small purulent patches. On each side of the petrosal process was a carious opening, which communicated with the carious cavity from which the diseased bone had been removed.

John Sidney, (65th,) was admitted for a fungoid enlargement of the testis, but for eight months before admission he had been suffering under various complaints, which successively appeared. At first he was attacked by a severe cold, which ended in hæmoptysis; then came on ear-ache of the right side; this was followed by a sense of numbness in the right hand, leg, and right side of head; and, last of all, came on very severe pain in the forehead. When first seen by the Doctor his face was slightly paralytic, the left corner of his mouth was drawn up, the right hand and leg were quite useless, his countenance was dull and vacant, his mind occasionally wandered, his pulse was feeble, and he was affected with frequent sickness. Leeches were twice applied to the temples, a sinapism to the stomach, and a blister behind the ears. Ammonia julep was ordered every six hours, and soda water was given for drink; but he gradually became weaker, the right side lost all sense and motion, and he sunk in the course of a few days. On removing the scull-cap, the brain felt very soft underneath the membranes, which in their appearance presented nothing unusual; but in making an incision into the left hemisphere, the arachnoid, pia mater, and external surface of the brain were all united into a thin wall, which when thrown back displayed the cerebral substance reduced to the color and nearly to the consistence of custard. When this liquid brain was removed to a level with the ventricles, a semi-transparent, pink-colored, oval tumour was found, the surface of which was firm and red-colored, while the interior was composed of a semi-transparent paste, which under the microscope seemed to be pervaded by many vessels, and had many of the properties of half-coagulated albumen. The right (a false type describes it as the left) was quite natural, nor could any other vestige of disease be discovered in any other part of the brain. Both lungs were so crowded with fungous tumors, similar to that found within the brain, that not more than one half of each was in a respirable state. The right testis was ten times its natural size, and composed of cysts of a fungous nature. The glands of the left groin were enlarged, and in their centres were small lumps of earthy matter.

"This is a case remarkable for the extent to which three very important organs had become the seat of fungous disease. It appeared to have begun in the testicle, then to have attacked the lungs, and lastly to have affected the brain. The symptoms of disease within the head had not shown themselves till about five months preceding his admission into the Hospital. On looking back to the history of the case, there is reason to believe that for several weeks after the commencement of the cerebral disease, the symptoms were confined to such as indicated irritation and pressure from the morbid growth; and that not till within three weeks of his death had the more active development of the fungoid tumour or some other circumstance paved the way for that mischief which terminated in the disorganization of a large portion of the brain surrounding the original disease." 129.

In the 66th and last case illustrative of this subject there was such general and extensive disease, as to render all diagnosis confused and unsatisfactory. The head affection was evidently the precursor of the rest, but how far the organs, which were subsequently attacked, were under the influence of the cerebral derangement, it is difficult to decide. A middle-aged man, who had lived very dissolutely, and had suffered severely from syphilis, was admitted into hospital with intense headache, accompanied by mental aberration and symptoms of general distress. About seven weeks before admission great pain had been felt in right ear, from which some bloody matter had been discharged with relief. The left became afterwards affected in the same way, but still more severely; and for seventeen nights before admission, his suffering and delirium were so great that persons were obliged constantly to attend upon him. Much unhealthy matter was discharged from his left ear with the effect of alleviating his sufferings in some degree; but the pain was still severe, his mind was incoherent, and he gradually sunk. A large sloughing abscess behind the left ear being cut down upon, the bone was found denuded of pericranium, rough and slightly discoloured. A little unctuous fluid lay between the dura mater and arachnoid. The brain was healthy, but the ventricles were distended with fluid. The lateral, petrosal, cavernous and circular sinuses were filled with dark, ill-conditioned pus, and the left side of the cerebellum exhibited a depression, which had been caused by a small collection of pus beneath the dura mater. The left jugular vein, until it joined the subclavian, was thickened, opaque, green, and filled with an unhealthy purulent fluid. In removing that part of the base of the scull, which contained the inflamed sinus, the cavity of the pharynx was opened, and a mass of lint in a most fetid state, sodden with pus, which seemed to have lain there for weeks, was discovered at its upper and back part. The cellular membrane of the mediastinum was filled with semipuriform fluid. A thin coating of lymph, and other effects of recent inflammation, were contained in the left thoracic cavity, and the substance of the left lung was studded with small sloughing abscesses, filled with dark olive green, putrid matter, and surrounded by thin cysts. The right lung was in a similar state; "but here the most extraordinary sphacelus had taken place of the pleura costalis, and of a quantity of false membrane which had been deposited upon it. The whole of the 4th, 5th, and 6th ribs presented internally one dark green mass, dreadfully offensive in smell." Some turbid serum lay in the pericardium, which was coated with a layer of recently-deposited coagulum. The abdomen was healthy.

In such affections of the ear as have been now described, the treatment upon which any hope of benefit can rest is antiphlogistic. In mild cases leeches, cupping, fomentations, emollient or astringent injections and aperients will be found sufficient to overcome the inflammation and to arrest the discharge. In some instances the disorder is so mild, that the discharge acts the part of a spontaneous and sufficient cure, by gradually depleting the inflamed vessels. But in other cases there is either such an original complication of disease, or the brain, from some peculiar susceptibility not easily explained, so strongly sympathizes with the local affection as to induce symptoms of derangement the most ferocious and formidable, that whatever treatment is pursued is almost equally unavailing. If the inflammation have been neglected on the outset, and have been suffered to make its way into the interior and delicate structure of the ear without check or diminution, the brain is almost certain of being implicated under any plan of management; and effects the most disastrous may be certainly anticipated. In these advanced forms of the disease the Doctor has found blisters the most useful remedies. Mercurial fumigations may be tried, and if the extent of the disease were clearly ascertained, acid injections might be of service. The Doctor thinks it probable that in some cases, in which the mastoid cells are diseased, the trephine might afford relief; but no example of its application has been adduced.

In few affections, we believe, does our treatment suffer more from false pathological views than in *hydrocephalus.* Hundreds annually die victims to groundless fear respecting debility, dropsical diathesis, obstruction and congestion. The presence of water is considered by many as essential to a pure hydrocephalic state of brain, as it is to hydrothorax, or ascites, or œdema, or any other form of dropsy; and the existence of this water is referred, not to previous action or inflammation, but to vascular relaxation, constitutional debility, or some chylopoietic derangement. The very essence of the disease is actually overlooked; and the whole attention is engrossed by a mere accident, which is as uncertain in its occurrence, as is hydrothorax after an attack of pleurisy. We may have pure hydrocephalus without a drop of water in the brain, and we may have abundance of water in the brain without one symptom of hydrocephalus. It is not the presence of water which is to be dreaded, as much as the previous action upon which the existence of this water depends. If this action be prevented from taking place, or be arrested ere it have gone too far, there will in general be no effusion; if this action be neglected it will often destroy life before effusion can occur; and effusion may take place without either aggravating, or altering the symptoms which preceded it. The term, therefore, by which this disease is designated, is most inappropriate and deceptive. It expresses not the thing signified, but a mere accident of the thing, and thus leads the practitioner to attach that importance to a consequence which is due to the cause. Water may be thrown out upon the surface, or into the ventricles of the brain, but in eight cases out of ten, we believe, before one drop is effused there is increased vascular action, if not decided inflammation, to be contended with. It is to this prior and causal state to which our treatment is to be directed, and not to the posterior consequential condition which may never occur, and adds but little, when it does occur, to the danger by which it was preceded. Debility is a phantom which the theorist alone can fear; effusion is a condition which the physician will rather prevent than remove; and hydrocephalus is a disease, which diuretics and tonics have seldom cured, when antiphlogistic measures have been unsuccessfully applied. It is gratifying to have Dr. Bright's support on a subject of such practical importance; for few have enjoyed more ample opportunities for observation, and few are better qualified, from their dispassionate coolness, dread of theory and love of truth, to trace these observations to their legitimate deduction. Hydrocephalus he decidedly regards as an inflammatory disease, and as only to be combated by anti-inflammatory measures. His recommendation, however, of antiphlogistic remedies is very judiciously guarded by restrictive cautions; lest from over-anxiety to escape one extreme the opposite error may be committed.

A boy, (Case 24,) aged 20 months, who had always had a trick of "butting with his head," was suddenly seized with severe pain in his head while asleep in his mother's arms. Leeches were twice applied to the temples, blisters were placed behind the ears, the bowels were excited by calomel and jalap, and a mixture with squill and almonds was employed. When the Doctor first saw the child his face was pale, his head was hot and in a constant state of motion, but never raised from the pillow; the pupils acted, there was no strabismus, and the pulse was 120. As the boy was teething the gums were lanced, a cold wash was applied to his head, the bowels were fomented, ipecacuanha was substituted for squill, and one grain of calomel, with three of magnesia, was ordered every four hours. On the next day the stools appeared green and curdled, the child made more complaint, and his hand was frequently carried to his head, more especially when asked to point out the seat of pain. Three leeches were placed on the temples, and two grains of subcarbonate of soda, with half a grain of calomel and five of compound chalk powder, were given every three hours. During the next day mercurial inunction was commenced, and the leeches were repeated. On the 12th a blister was applied behind the ears; and with occasional blistering afterwards, the employment every eight hours of an antispasmodic mixture, stimulating pediluvium and mercurial friction, the treatment was pursued until the evening of the 17th, when the child died. From the 11th until his death the principal symptoms were convulsions, restlessness, and occasional insensibility. "On raising the dura mater all the larger veins on the surface of both hemispheres, running into the longitudinal sinus, were seen round and hard, quite filled with yellow-coloured coagulum, as if injected with wax; while the whole vertex was covered under the membranes with extravasated blood."

The longitudinal sinus was also occupied with a firm coagulum, which was composed almost entirely of fibrin. On cutting through the brain a little above the ventricle, a number of small round red spots were discovered, which a microscope revealed to be small extravasated coagula; and the outside of the anterior lobe of the right hemisphere was so softened, as to break down with ease upon the pressure of a finger. The ventricles were distended with fluid.

It is the opinion of Dr. B. that depletion was carried too far in this instance, and that the constitutional debility which ensued accounts for the tendency to coagulation, which existed in the blood. That by weakening the powers of life we promote coagulation of the blood is a physiological fact, which has been long acknowledged; and that by carrying depletion to an extent incompatible with the stamina of the constitution, we may give rise to such debility is equally ascertained; but when it is considered that in the present case 14 leeches only were applied, and, beyond one or two small blisters, no other measures of a depleting nature were adopted, some may have some difficulty in understanding how treatment so moderately active, in an attack so sudden and so ferocious, could have produced the result which is here ascribed to it. This boy had always been remarked for striking his head forcibly against objects which lay near him, proving that some cerebral disurbance had existed long before he became an object of treatment; and when it is known how insidiously and how long even inflammatory disease may be working its effects within the brain, one would rather ascribe any debility, which may have marked the close of this case, to its lengthened effects upon the constitution, than to the moderate measures which were called in to combat the symptoms of its last stage.

A boy, (Case 168) of 7 years of age, having a large head with rather a contracted chest, and betraying considerable aversion to mental exercise, became exceedingly irritable, sick at stomach, and complained of constant pain at the fore part of his head. By and bye he began to dislike the light, he grew drowsy, had strabismus of the right eye, and was occasionally incoherent. When it became obvious that his brain was disordered, his mother recollected that of late he disliked to hold his head low, or do any thing which required stooping. After sinking under these symptoms he was examined, and the sutures gave way a little on the scull being raised. The veins of the arachnoid were distended, the convolutions of the brain were flattened, and when an incision was made into the brain at the usual depth, to remove the top of the hemisphere, the lateral ventricle was opened and a quantity of clear fluid escaped. Not less than from six to eight ounces of serum occupied these cavities. Several spots of ecchymosis were discovered in different parts of the brain, but more especially in the roof of the posterior extremity of the left ventricle, where the cerebral tissue was much softened. This softening, however, was confined to the medullary part; as the cortical covering was unchanged. Several tubercular formations were discovered in the pleura, lungs, omentum, and peritoneum.

An active child, 10 years old, (Case 18th) began to complain of indistinct and double vision, of pain in the temples, drowsiness and cerebral oppression. These symptoms were followed by squinting, listlessness, vacancy of countenance and mental aberration. Her drowsiness increased, her articulation faultered, and she walked with an indecisive step. Her head was shaved and cold was applied; eight ounces of blood were taken from the nape by cupping, and calomel was given every six hours with soda and comp. chalk powder. By the next day her head was easier, and she no longer saw double; but her urine was scanty and her pulse kept up at 108. Ten leeches were applied to the temples, an opening medicine was given, and the powders were repeated. On the day after the head was free from pain and oppression, but there was some obliquity of vision, and the pulse had risen to 120. The same number of leeches were again ordered, and the calomel was continued. Seven days after she had been subjected to this treatment her mouth grew tender, so that the mercury was omitted; but blisters were applied between the shoulders and behind the ears, the purgatives were persevered in, and in about a week afterwards the squinting had disappeared, and, excepting weakness which prevented her from walking firmly, no other vestige of complaint remained. Half a grain of calomel was notwithstanding continued twice daily.

The suddenness of the attack, viewed in connexion with the moderate degree of head-ache which was here complained of, throws an aspect of uncertainty over the real condition of the brain in this instance. The symptoms were purely hydrocephalic, and the treatment was mainly directed against effusion. But whether serum could have been extravasated in so short a period, and with such apparently trifling excitement, at least to an amount sufficient to produce such very great cerebral disturbance, is somewhat questionable. Mere congestion of the vessels of the head, as the Doctor very justly observes, will occasion hydrocephalic symptoms as certainly as effused lymph, or serum; and, although we feel no difficulty whatever in believing that serum extravasated within the head may be absorbed, notwithstanding our inability to discover many absorbents in its texture, yet, from the rapidity with which the disease in the present instance made its appearance, from the uniform good health of the patient previously, from the great relief always afforded by the removal of blood, from the continuance of strabismus after the mercury had produced its effects upon the system, and from the speedy disappearance of every symptom, it would not appear unreasonable to conclude that vascular turgescence, rather than strong inflammatory action on the one hand or serous effusion on the other, was the pathological state on which the symptoms in this case depended. How hydrocephalus may be accidentally developed in a predisposed habit by various sources of irritation, as injuries inflicted upon the head, tumours imbedded within the substance of the brain, teething and such causes, is well shewn by the following cases.

A girl 7 years of age, (Case 15th) of a scrophulous habit, injured the back of her head by falling on the pavement. She was at first stunned by the blow, ten days afterwards objects began to appear double, and six days before her death she was seized with convulsive fits, in which her speech was lost, which frequently recurred. Towards the end of her illness the voluntary muscles were especially affected by these convulsions. She rolled from one side of the bed to the other, she threw herself from the bottom of it to the top, and although her mind was quite undisturbed, these motions were so violent and so disobedient to control, that the greatest watchfulness was necessary to obviate their consequences. On inspecting the head after death, the dura mater was turgid, the convolutions of the brain were smooth, its veins were large, but no fluid was effused upon it. The substance of the brain presented nothing unhealthy. The lateral ventricles contained one oz. and a half of clear serum, the smaller ventricles were also full, and a little fluid lay at the base of the scull.

A boy, (Case 70th) five years and a half old, of an irritable temperament, was seized without any evident cause, when he was two years old, with convulsive fits, which gradually became more violent, and at length terminated his life. These fits were at first separated by considerable intervals, were attended with screaming and followed by sleep; but they afterwards became very frequent, and issued in palsy of both the upper and lower extremities. He swallowed with difficulty, but his appetite was good and his kidneys and alimentary canal discharged their functions with unimpaired vigour. Throughout his illness he seldom complained of his head; the last four months of his life he spent in a state of stupor, and he seemed to have lost his sight, but without strabismus, not long before his death. A younger sister of this child is now affected in a similar manner, and his grandmother on the mother's side has laboured for the last four or five years with hemiplegia of the left side. This little boy had been salivated and blistered along the spine, but little relief had followed any remedy save leeching. The dura mater was found thick, the arachnoid spongy, unusually red and closely lining its internal surface. These membranes were distended with fluid, which had by its pressure separated and contracted the convolutions of the brain. Many large veins seemed to lie on the surface of this fluid, and when it was removed the brain appeared small and covered with venous vascularity. The longitudinal and lateral sinuses were filled with a soft red coagulum, the pia mater was attached to the surface of the brain, and the cortical substance was so soft as to separate with ease from the subjacent medulla, which was proportionally firm. About one ounce of serum occupied each ventricle, a little was deposited at the base, and on cutting into the lower part of the middle lobe on the left side, a yellow tubercle of the size of a large pea was detected at the bottom of one of the convolutions. The cerebellum was pale, but the membranes were quite healthy. Whether there were any similar tubercular deposits in the other organs we are not informed; but it will scarcely be conceived that the extensive mischief, here done to the membranes and cineritious substance of the brain, had much more connexion with the small isolated tubercle which lay in the middle lobe, than it had with the production of the general symptoms. The following case, which probably owed many of its phenomena to dentition, bears a very striking similarity to that now given.

(Case 19th.) A little girl, who was only three years and nine months old at the period of her death, began to cut her teeth at the age of ten months, and during that process suffered severely. This first little illness once over, she gained strength rapidly and could run about at fifteen months; but before her second year had been completed, she was seized with convulsive fits which were at first referred to dentition. The intervals between the fits, however, were spent in peevishness, her limbs gradually became stiff and rigidly extended, and her mind betrayed symptoms of approaching infirmity. At length, for an entire year before her death, she was so stiff as to be quite incapable of motion. The feet were stretched, the thumbs were drawn into the palms of the hands, deglutition became difficult, the bowels inactive, and vision, at first impaired, was ultimately lost. By unremitting care she was kept alive in this state for some time; but after passing three very restless nights, she was attacked with convulsions, under the effects of which she rapidly sunk. In removing the scull the dura mater was wounded, and about five ounces of limpid fluid escaped; but whether it lay beneath, or above the arachnoid it was impossible to ascertain. The membranes were generally healthy; the brain, when compared with the size of the cranium, appeared much contracted. On cutting into the cortical substance it was found thick and pulpy, but the medullary part was contracted, and so hard as to give to the finger when divided the sensation communicated to it by the knife on cutting cartilage.

"The hardness of the whole medullary portion was such, that by scraping with the handle of the scalpel, no impression whatever was made by a degree of force which would have scraped in pieces an ordinary brain, but the scalpel passed over the cut surface as it would over a membrane; towards the edges the hardness suddenly increased, so that there was a margin round the whole, more white and prominent than the rest. When a portion of the brain was pressed between the fingers it gave a very peculiar sensation, the cortical part yielded readily, and the convolutions of medullary matter were felt beneath like an irregular hard body; and by pouring a little water on the outside and gently rubbing, the whole cortical portion was removed, leaving the medullary convolutions exposed, quite firm, in ridges like the rugose mucous surface of the stomach in some animals; it was perfectly firm, allowing to be freely rubbed and washed. Some portions of the brain were rather harder than others, but all parts on being cut, gave the idea of a wax model rather than brain. The ventricles were moderately distended, and retained their open form, as if moulded in wax; the membrane lining them was thick, so that in a section it seemed as if they were lined with a semitransparent membrane, of a line or two in thickness. The plexus choroides natural.

The cerebellum was pallid, and the cortical portion soft; and when cut into, the corpus rhomboideum with the surrounding medullary matter was found completely softened, but round the outside the brain was nearly as hard as in the cerebrum, so that on scraping very gently with the handle of the scalpel, a cavity with pretty firm parietes was apparent." 45.

The nerves at the base of the brain, especially the optic nerves, cut like soft cartilage, were elastic and stood quite erect. The medulla oblongata and the small portion of spinal cord removed with it partook, though in a less degree, of the general hardness. The non-intermixture of paralysis in this case with the convulsive symptoms, Dr. Addison, under whose care this patient had been placed, was inclined to ascribe to the external position of the effused fluid; and Dr. Bright thinks that this view receives corroboration from the softened condition of the cortical layer, and by a case afterwards detailed, which was characterized by convulsions without palsy, and in which examination after death discovered a coagulum of blood deposited in the cineritious substance of the brain. Still, however, it must be admitted that large quantities of serum have been found effused between the membranes, without having induced during life such tonic spasms, and in the 170th case already given, where the cortical texture of the brain was in the most complete state of softening, in addition to its having had a quantity of fluid between its membranes, we find very general paralysis without any convulsions after the first stage. How far the very condensed and indurated condition of the medullary texture depended upon pressure of the surrounding fluid is an interesting question, the solution of which might shed some light upon phenomena at present too obscure to warrant any thing stronger than conjecture. The 26th, 27th, 28th, and 29th cases are so highly interesting, as to merit separate notice in the Periscope.

The space, which the Dr. allots for the treatment of acute hydrocephalus, is small; but in this, as in almost every other instance, he confines himself to a few general observations, leaving the reader, who looks for more minute detail, to trace it through the cases with which he so amply illustrates both its nature and management. As some degree of inflammatory action, he observes, usually exists, it is upon the readiness with which this action is discovered and subdued, that our only hope of cure can be founded. He cautiously, however, warns against the adoption of extremes, and believing that mere irritation not unfrequently exerts a prominent influence even in the earliest stages of the disease, he reprobates the repetition of small bleedings upon the appearance of every trivial symptom, as calculated to depress the powers of life, more than the principle of the disease. In many such cases nourishment and gentle sedatives will be proper. He has seen five drops of laudanum, given three times daily to a child of two years old, extricate it out of most alarming symptoms; but as a general rule he would prefer commencing with a smaller dose, and regulate the quantity according to the effects produced.

We believe it is always a difficult diagnosis to distinguish severe irritation from mild inflammatory action; but in diseases of the brain the great and many peculiarities of the texture affected render this difficulty ten-fold greater. The source of sensibility is itself the seat of the disease, and inflammatory action may be masked by symptoms of the most opposite character, just as it is chronic or acute, in the first or last stage of its progress, in a sanguine, scrofulous, weak or robust habit. The line, therefore, which separates what we call simple irritation from slight inflammation of the brain, probably exists in every case but is certainly seen, we apprehend, in few. These conditions pass and repass so insensibly into each other, and are so frequently combined, that they are reciprocally each other's effect and cause, and any therapeutic principles, which are to be based and directed upon a distinction so fluctuating, require to be adopted with great caution and carried into operation with enlightened watchfulness. But, as we shall have another opportunity, in the course of our review, to recur to this subject, we go on with our author to observe that, in addition to such cases as depend upon inflammation and irritation separate or combined, there are others in which congestion appears to be the morbid state which is productive of distress. This congestion may depend upon some original disproportion between the activity of the arterial and venous systems, or upon some local obstruction; and our treatment must be regulated accordingly. Where inflammation exists, general and local bleeding and the continued application of cold are necessary. Where irritation prevails, depletion must be more sparingly, if at all, employed. And in congestive hydrocephalus blisters to the nape, cold dash occasionally to the scalp, and cupping on the temples, if the strength permit it, will be proper. The following observations on the use and action of mercury are well worthy of attention.

"The employment of mercurials in hydrocephalus has long and justly been considered of the utmost importance; but I am persuaded that the mode in which they have been used, and the object which has been proposed by their administration, have both been often erroneous. Calomel has usually been given in frequent doses, with a view of acting upon the liver, and producing repeated green evacuations; and this by some has been considered the proof that the calomel is acting favourably, whereas by others, when the green stools have appeared, they have been assumed as the proof that more calomel was required to clear away the vitiated bile. The fact seems to be, that such stools are produced by the action of the calomel; and as far as can be inferred from the symptoms of irritation with which their occurrence is usually accompanied, and from the appearance of the intestines of children who have died while calomel was producing this action on the liver and bowels, there is reason to believe that they have been a source of much irritation to the intestinal canal; for although it is often quite impossible in children during life to ascertain the exact cause of irritation upon which the symptoms depend, yet on dissection we find that the bowels are in an irregular state of contraction, and the masses of green feculent matter are distributed amidst the unequal contractions in such a way as to show plainly that they have been a source of irritation, and not unfrequently numerous portions of the intestines have been found in a state of intus-susception. To those who are acquainted with the influence which irritation of the mucous membrane exerts on the brain in the febrile attacks even of adults, it will be quite unnecessary to urge the probable effect of such irritation on the delicate nervous system of children in whom the brain is already rendered acutely sensible by disease: and by those who have witnessed the convulsions of children caused by griping, it will also be allowed that irritation of the muscular fibres may exert a powerful influence; and I shall have hereafter an opportunity of showing that inflammation of the external or peritoneal coat is likewise capable of producing intense cerebral irritation.

In the administration of calomel, then, it is a great object to produce as little irritation as possible. Mercury acts in this disease on different principles: in the commencement it is the most powerful and effectual means we possess of reducing the inflammatory action set up in the membranes of the brain, thus preventing the effusion of serum; and in the more advanced stages it may assist in producing absorption. It will likewise act on the secretion of the liver, and either by itself or in combination, assist in removing any feculent matter which might prove the casual source of irritation. If the calomel is suspected of irritating the bowels, no more rational proceeding can be adopted than to combine with it an opiate, as we always do in adults when we give it with a view of producing a favourable change in the vascular action of inflamed membranes: it is true that the use of opium in children so tender as those who are frequently the subject of hydrocephalus is not without its hazard; but this may be obviated by caution; and in so fatal and unmanageable a disease something should be risked if a great good is to be hoped. One thing is always to be borne in mind in our treatment of this disease, that, while a due regard to the delicacy of the frame on which we are acting should inculcate prudence in the use of depletion, and caution in the administration of such remedies as may induce excessive irritation; the recollection of the extraordinary recoveries which all have witnessed, after every rational ground of hope seemed to have vanished, should always prompt us to assiduous perseverance in the use of remedies as long as life remains." 74.

Dr. Bright has found it convenient to treat of chronic hydrocephalus in that section of his work, which is appropriated to the effects of pressure upon the brain; but we prefer noticing this subject in the present place from our belief that, if there really be any meaning in the separation of this malady into chronic and acute varieties, the most material point in which they differ is, not the nature, but degree of action on which both depend. In acute hydrocephalus the symptoms are more severe, the action is more phlogistic, the disease is more rapid, and there is less time, while there are more indications, for treatment. In the chronic form the vascular excitement is moderate, the course of the disease is tedious, the constitution sympathises slowly, and active measures are seldom indicated, and more seldom borne. Still, however, if the term hydrocephalus be continued to express this morbid action, which is common to both the acute and chronic forms, these varieties cannot be considered as essentially differing; nor can the one form be combated by remedies totally inadmissible in the management of the other. From some modifying cause, which is not easily ascertained, the action, which precedes effusion in the chronic form, is so subacute and so insidious that it often deluges the brain, distorts the sutures and distends the scull, without being betrayed by any unusual activity of pulse, acuteness of pain, or cerebral disturbance. The water either accumulates so slowly as to diminish the effects of pressure by their gradual development; or there is an unnatural passiveness of sensibility, upon which ordinary impressions fail to operate their ordinary effects. The brain has been often known, in the last stage of this disease, to bear, without manifesting serious symptoms of disturbance, a degree of pressure which was equal to separate the sutures and forcibly expel the fluid for which there was no longer room within the cavity of the scull. And where the tendency to effusion has ceased before the closing of the fontanelles and sutures, patients live many years in tolerable health, although during all that time the brain is subjected to the pressure of many pounds of fluid. When, however, the disproportion between the exhaling and absorbent functions has become too great to have its injurious influence counteracted by expansion of the head, excruciating pains, convulsions, paralysis and insensibility rapidly succeed, unless means are found to extricate the brain from such inordinate and increasing pressure. The following case will forcibly illustrate how long life may be protracted and mental function preserved under the constant operation of cerebral pressure.

A man, aged 29, (Case 205,) who had laboured under chronic hydrocephalus from childhood, was admitted on the 1st of December, 1824, into Guy's Hospital. When born his head was very little larger that natural, but it had a pulpy feel, and was apparently almost destitute of bone. A fortnight after birth, however, it began to enlarge, and continued to do so for five years; after which it made little if any progress till the period of the patient's death. While a child his health had been good; when about three years old he had frequent attacks of epistaxis; at six, when he went to school, he was able to walk alone upon a level surface, and although he could soon read well and write tolerably, his writing was discontinued in consequence of headache, which was occasioned by the act of stooping. His appetite was ravenous, but he was weak and shook as he walked, and when a candle was held behind his head, his cranium appeared transparent until he reached his 14th year. Fits of an epileptic nature came on when he was 23; the bones of his head closed when he was 27, since which he had shooting pain in the vertex; and in the Summer of 1823 an abscess formed in his ear, which relieved his head. When admitted into hospital he appeared in tolerable health, his countenance was not wanting in intelligence, he was near-sighted, his other senses were acute, the faculties of his mind were possessed of tolerable energy, his passions were irritable, he was fond of society but had no sexual desires, his head ached occasionally, when he stooped it became giddy, and when he moved violently he complained of a sharp pain in his forehead. His voice was hoarse, his bowels were costive, and he was unable to lie on his right side. A few weeks after admission he caught cold, febrile symptoms appeared, he lost his appetite, diarrhœa came on, and he died on the 24th of February, 1825. On dissection the upper part of the cranium was found occupied with a clear fluid, which was contained only within the dura mater, and the brain lay flattened and depressed at the base of the scull.

"What we saw looking like the flattened superior surfaces of the two hemispheres, was in fact the two lateral surfaces, which in health are opposed to each other through the centre of the brain; these had been separated and thrown back by the pressure of the fluid which, having first accumulated in the lateral ventricles, had forced a preternatural opening on one side of the corpus callosum, which, together with the whole fornix, had been nearly obliterated; and as the fluid increased, the hemispheres were gradually brought down to the base of the skull. The right hemisphere was quite flat, but the left was raised into a point at the side of the skull. About one pint of fluid was found in the ventricles, and probably, six or seven pints externally; for when the skull was completely freed from its contents, both solid and fluid, it would hold ten pints of water. The ventricles were so much disfigured by the fluid within, as well as by the peculiar position the brain had assumed, that it was with difficulty the various parts were discovered; to add to which difficulty,—as it was thought advisable to procure drawings of the appearances,—two or three days elapsed before we could pursue the dissection more particularly, and then many of the finer parts were rendered indistinct by exposure. The cerebellum was flattened and vascular." 433.

The longest diameter of the scull measured 32 inches and one quarter; the distance between the articulations of the lower jaws over the head was 21 inches; from the root of the nose to the posterior margin of the foramen magnum the space was 23 inches and three-eighths; the antero-posterior diameter was 10, and the transverse diameter 9 inches. The scull was very thin, but ossified; the interior surface of its thickened portions had a worm-eaten appearance, and between the regular bones there were ossa triquetra of various size and form, united by sutures.

In the 202d case an instance of chronic hydrocephalus at birth is given by Mr. Hargraves of Tunbridge Wells. Mr. H. had been called in to attend the confinement of a woman, who had been under a midwife's care for three days. He found a portion of the scalp protruding at the os-externum, and by carrying his finger up into the pelvis he discovered a rupture in the anterior surface of the uterus, where it is connected with the bladder, upwards of four inches in length. Finding it necessary to deliver the woman by assistance, he punctured the head and discharged from it three or four pints of fluid; after which no further interference was found necessary to terminate the labor. The child appeared perfect in every part, save the back and head: it was unusually large, and seemed to have been dead twelve or fourteen hours. Dissection ascertained that the perforator had gone through the scalp only, that the scalp was separated from the head to a great extent; that a good deal of the fluid which escaped was exterior to the cranium, and that the brain was natural both in size and structure.

In a letter to Dr. Bright, Dr. Bostock has given to the world some chemical observations on the fluid generated in hydrocephalic disease, from which it appears to consist, in the thousand parts, of water 982·6, of albumen 6·, of muriate of soda 7·, of soda 1·4, of urea and osmazone 3·, and a trace of sulphuric acid, lime, and potash. As the presence of urea has not, we believe, been pointed out by any previous analysis, it may be well to give in his own words the plan by which it was obtained.

"After digesting the residuum (says Dr. Bostock) procured by evaporating the entire fluid in successive portions of tepid water, evaporating the solution thus obtained, treating the residuum with alcohol, and evaporating the alcohol, a second residuum was procured, which I describe as 'deliquescing; by a gentle heat it 'was reduced to a semitransparent substance, partially brittle and partially viscid, 'exhibiting somewhat of a crystalline structure, but no distinct form could be 'perceived. It had a pungent taste: a drop of nitric acid added to a portion of 'it produced considerable effervescence, and there was an appearance like the 'pearly scales of the nitrate of urea. A quantity of nitric acid, diluted with an 'equal bulk of water, was added to the residuum; there was considerable action; 'heated over a lamp the whole was dissolved; with extrication of nitrous va'pour; I did not perceive any nitrous gas. Set by to cool, after it had been 'gently heated, so as to diminish the whole by about one-third, a quantity of 'crystalline scales appeared round the edges. The next morning the whole was 'liquid, having attracted moisture from the atmosphere. It was now gently 'heated to dryness over the lamp; the scaly crystalline mass again formed; I 'should not have known it from nitrate of urea. By testing it with the solution 'of muriate of lime, it was found not to contain oxalic acid.'" 441.

Some of the peculiarities, which we have noticed in the nature of this chronic form of hydrocephalus, and others of an equally peculiar character, have rendered its treatment very difficult. Some, regarding it as purely a disease depending on debility, have confined their curative indications to strengthening the general constitution, exciting the absorbents, or treating it entirely as a mere dropsical affection. Mercurials have been tried by one party, cathartics by another, blisters, issues and setons by a third, diuretics and tonics by a fourth, while others, disappointed by all these plans, have sought for relief from surgical assistance. From the trials already made it is quite certain that puncturing the head may be repeatedly performed without producing death, and the issue of some of then has even been encouraging. In 1778, Dr. Remmett, of Plymouth, operated on a child only two months old. The head was punctured five times in the short space of four weeks, and no less than eighty ounces of fluid were drawn off. The child, however, died 17 days after the last operation, and the whole cavity of the cranium was found full of a pellucid fluid, of which above two quarts were collected. In 1817, Dr. Vase, of Liverpool, punctured a hydrocephalic head four times, and drew off 32 ounces of fluid; but, although great relief followed the operation, the patient ultimately sunk. Mr. Callaway has since been equally unsuccessful at Guy's Hospital; and perhaps the cases of Dr. Conquest, which have been lately laid before the public, are the most favorable to this operation which we yet possess. Dr. Conquest has operated repeatedly, and in at least two instances with apparent success. At the first operation upon one of his patients 12 ounces of fluid were withdrawn, and at the second 18 ounces. The head has since closed, and the child is stated to be apparently well, although it had been previously much afflicted with convulsive fits. We cannot refrain from expressing ourselves considerably sceptical on the value of this operation. It may, no doubt, give temporary relief in some instances, and may prolong for a few months, or if you will years, a miserable existence; but we strongly apprehend that the state of brain, accompanying such extensive effusion as requires tapping, holds out but little prospect of ultimate success to any form of treatment. Dr. Bright, however, is rather favorable to the operation; and as it can always be easily employed after every other plan of cure has been tried and failed, it is a dernier resort which may foster hope without diminishing in the least our other chances of recovery.

"The apparent success which has attended one or two cases, holds out a slight encouragement to a more extensive trial of this most doubtful remedy. There is no doubt that many cases will fail; for in some the tendency to pour out fluid continues unabated, and between each successive operation the head rapidly increases: but if, fortunately, as sometimes in the operation of paracentisis of the abdomen, the tendency to accumulation should have ceased, either from the effects of remedies, or from some local change depending on the abstraction of the fluid, and if the cerebral disorganization should not be totally irreparable, a cure may be effected: still, however, it remains to be proved to what extent such cures will be satisfactory as to the future mental condition of the child; and probably a very small proportion will even apparently succeed." 428.

Having now traced the effects of disease from the skin to the scalp, from the scalp to the scull, and from the scull to the membranes, the effects of inflammatory and other morbid actions upon the texture of the brain itself occupy the attention of the Doctor, and under the two heads of abscess and ulceration he cites a few interesting cases. Had opportunity allowed, it would have made his series more methodical and complete, if the Doctor had given us some instances of phrenitis before the inflammation had terminated in either abscess, or ulceration. He treats largely on softening of the brain, but not as an effect of inflammatory action; and he is copious in his illustrations of the results of pressure from turgescence and effusion. But that intense vascularity, which the brain presents in the early stages of inflammation, he has not exemplified by any well-marked cases. Two very distinct cases of superficial ulceration of the brain, three instances of encysted abscess, and two examples of extensive cerebral destruction somewhat analagous to sloughing abscess in other textures, are recorded. The matter formed in cerebral abscesses is very different from the ordinary purulent fluid, which is elsewhere secreted in such instances. Indeed, the author has been induced to conclude that "from some peculiarity in the structure of the brain true pus is scarcely ever formed, unless when some tubercular matter has been previously deposited, or a cyst formed; in which cases even it puts on a tenacious mucous character."

(Case 72d.) A man of advanced age had labored for some time under dyspnœa, and other symptoms generally attendant on hydrothorax; and when admitted into hospital he was in a state of drowsy stupidity, he passed his evacuations involuntarily, and was unable to leave his bed. Blisters were applied to the chest, and some diuretics were given. His mind was very much confused, he was extremely noisy during night, and towards the close of his illness his speech became thick and indistinct, and there was obvious loss of power in the right cheek. A thin layer of blood was effused upon the posterior part of the left and the anterior surface of the right hemisphere. This layer varied in thickness from that of paper to a shilling, and was so attached to the under surface of the dura mater as to make it doubtful, when peeled off, whether it were enclosed within a distinct membrane. The under surface of the anterior lobes of the brain contained three or four patches of irregular ulceration, about as broad as a shilling, and the eighth of an inch in depth. The vessels of the base contained a few cartilaginous deposits; a curious black, carbonaceous-looking matter lay on the surface of the medulla oblongata, which seemed to have been the remains of a former effusion of blood under the arachnoid. The right lung was so diseased as scarcely to contain respirable texture. The left lung was similarly, but less affected. The left ventricle of the heart was hypertrophied, the aorta dilated and diseased, with cartilaginous deposits along its whole course. Several pints of yellow serum lay in the abdomen; the spleen was very small; the kidneys were contracted, externally scabrous and internally mottled with the white deposit. A double hydrocele in scrotum.

The paralytic symptoms in this case appeared only at the very close of life, and were too trifling to lead to the suspicion that much disease existed within the head. The Doctor had all along ascribed the cerebral disturbance to the difficult transit of the blood through the lungs, and the disease was altogether too complicated to construct any precise diagnosis upon its symptoms. In the second case of ulceration, the effects of this peculiar form of disease upon the general character of the affection are also very obscure, if at all perceptible.

(Case 71st.) A healthy man, nearly 60 years of age, scraped his left temple and eye-brow by a fall. Simple dressings removed the urgent symptoms, and it was only after having travelled in wet clothes three weeks afterwards, that any severe signs of cerebral disturbance were exhibited. He was bled from the arm, leeched on the left temple, and took remedies for the alleviation of his febrile symptoms. The injured part becoming puffy it was freely cut down upon, and poultices with fomentations were assiduously employed—although this treatment obtained immediate, it did not furnish permanent relief; he was very delirious during night, erysipelas came over the affected part, and he ultimately sunk. The bone was denuded to a considerable extent, and around the denuded part was very brittle. Opposite the separated portion of pericranium the dura mater was still attached, but a deposit of organized membrane, as thick as the dura mater, had formed between it and the external surface of the scull. The surface of the brain was covered with a serous effusion, and on the edge of the anterior lobe of the left hemisphere, close to the falx, was an oval depressed ulcer of the size of half a hazel-nut, containing a small quantity of white puriform fluid.

The first two cases of encysted abscess within the brain which are detailed are extremely similar, and illustrate in a light the most forcible the insidiousness and perplexity of all cerebral symptoms. In the 73d case there was abundance of proof that the head was seriously disordered; but whether the cause of disorder lay in the membranes, or medullary structure; whether its nature was effusion, suppuration, abscess, or mere inflammation —it was, perhaps, impossible to decide. The author believed that effusion was going on as the result of preceding inflammation, and accordingly he carried the use of mercury to the extent of affecting the mouth, but no relief was obtained; and the post-mortem appearances sufficiently explain the cause.

A young man had been afflicted with pain in head for three weeks before his admission into hospital. It frequently made him sick, he sighed often, rolled his head about, and cast up his eyes; but was unable to point to any particular part of the head as its principal seat. He had been bled from the arm, and then cupped on the nape, had cold applied to the scalp, and took hyd. c. creta every eight hours. He admitted that this treatment had somewhat diminished his suffering; but that his head-ache was still considerable. His nape was again cupped and blistered, one grain of calomel was given every six hours, and a saline mixture. On the next day the pain became intermittent, he still rolled his head, was occasionally delirious, and said that he was sometimes nearly blind. The pulse, which had at first been 84, now fell 20 beats, but the sighing was as frequent as before. Sixteen leeches were placed on the temples, and the other medicines were continued; but, although his mouth was becoming sore, he was evidently sinking, and the mercury was displaced by a dram of the tincture of Serpentaria every four hours. Coma gradually came on, he lay with his head bent down and his knees up, and in two days afterwards he died. The dura mater was vascular, the falsiform process was wanting, except in the posterior part, the arachnoid was very dry, the cerebral convolutions on the centre of the middle lobe were flattened, the two hemispheres adhered where the falciform process was deficient, the substance of the brain was healthy, the ventricles contained several drachms of colorless fluid. On raising the middle lobe off its site on the petrous portion of the temporal bone, a large part of it was found discolored and in a state of apparent suppuration. The dura mater, corresponding to this disease, was slightly discolored, thickened, and at one part detached from the bone, where a little bloody coagulum lay between them.

"Cutting into the diseased portion of the brain, it was obvious that the external portion was soft and discoloured, but separated easily from a cyst beneath it, which was of the size of a hen's egg, and contained a quantity of greenish-yellow pus; some of the more fluid part escaping, but the greater part being of a thick and rather ropy consistence remained, when the cyst was fairly divided through the centre; and part of it was attached in flakes to the cyst, so as to require to be forcibly scraped by the handle of the scalpel, appearing to be the inner surface of the cyst itself in a state of softening. The cyst was as thick and nearly as tough as a piece of wash-leather; but owing to the time necessary to execute the drawings, it was nearly three days after the removal of the cyst before I could carefully examine its structure: at that time in many parts no vessels could be discovered, but towards its upper part many vessels of considerable size might be traced, the trunks of which seemed to run along the internal surface, and their fine extreme division to float in the curdled pus with which the cyst was lined." 152.

The 74th case is so very similar to the one now recorded that it will be quite sufficient to refer to it. In the 75th case a young lady went through an attack of continued fever without any marked local affection, and during convalescence a discharge of blood and pus took place from the nostrils; after which cerebral symptoms appeared, and she died with evident indications of pressure on the brain. A table-spoonful of thickened pus was found between the dura mater and scull-cap, opposite the left tuberosity of the os frontis. The lining of both frontal sinuses was extensively ulcerated, and from the left an opening sufficient to admit a crow-quill led into the cavity of the cranium. The left hemisphere was so flattened as to occupy the greater part of the corpus callosum, and when cut into its anterior lobe discovered a cyst, which contained three ounces of pus and mucus. The father of this young lady, it appears, had a similar discharge from the nose, which was removed by fumigations with cinnabar.

Not only are symptoms indicative of cerebral disease peculiarly equivocal, as respects the nature of the action which exists; but they are also a most uncertain measure of the extent of danger which this action causes. We have already seen many striking illustrations of the accuracy of this fact, and the two cases which follow are highly interesting with the same view.

(Case 77th.) A child, 20 months old, fell with a cup in its hand, and an angular portion of the broken cup penetrated the posterior part of the frontal bone on the right side. The greater part of the fragment was extracted with some difficulty, and some hæmorrhage followed. A poultice was applied and minute pieces of the cup came away in the discharge. In about a week after hernia of the brain came on. Lime water, simple dressing and pressure were employed, and the wound for some time improved; but pulsation at length appeared, fever ensued, the granulations grew glassy, the discharge became unhealthy, the appetite declined, convulsions came on and death followed. The edges of the external wound adhered to the pericranium, which for some extent was separated from the bone. In removing the scull, a quantity of purulent matter exuded from the herniated portion of brain, which was contained in a cyst. The right hemisphere was externally much unfolded, and in the right part of the centrum ovale an abscess, which freely communicated with the right lateral ventricle, occupied a large space in the anterior and middle portions of the brain. From 10 to 12 ounces of sero-purulent fluid were contained in this abscess and ventricle, and a piece of bone, which the cup had thrust before it, was found in the anterior end of the abscess.

A middle-aged woman (case 78th) was confined with her second child on the 27th of January; on the 4th of February a tertian ague came on; and on the 11th she was seized with an epileptic fit. She was bled to 12

ounces and the blood was buffed. A second fit occurred, but in a more moderate form. Her head was shaved and kept cold, rest was enjoined, she got four grains of blue pill and some tartar-emetic wine every four hours, and she was cupped to eighteen ounces on the nape. Pain was now complained of in the muscles of the right side of head, and she kept her hand very generally on her left ear. On the 12th, the left angle of the mouth was somewhat retracted, the countenance was more vacant, and the temperature of the right side of head was evidently inordinate. Leeches were applied to the temples, and a sedative pill, which had been given the night before, was repeated. On the 13th there was a little more cerebral disturbance, and the left arm seemed weaker than the right. Fifteen leeches and a sinapism were ordered, together with a blister to the nape. On the 14th and 15th she was better, and during the 16th and 17th she was very much improved. On the 18th she passed a restless night, and complained of a feeling of distension on the right side of head, attended with uneasiness; and on the 15th convulsions and perfect inconsciousness came on, which sinapisms to the feet, bleeding from the arm and other measures were unable to remove. The dura mater was thickened and injected. The left hemisphere was rather vascular, but in other respects healthy. The surface of the right hemisphere was still more vascular, and its substance was almost wholly occupied by an abscess, which opened upon the surface by a hole, surrounded with coagulable lymph, opposite the meatus auditorius externus. The cerebellum was quite healthy. As to the origin of this extensive disease no cause could be conjectured, excepting a slight blow received on the head during the previous Autumn, which was productive of some temporary pain, but was insufficient to attract much notice even at the time of its occurrence.

(Case 80th.) A negro had been attacked while at work two days before his admission into hospital with palsy of the right side. Four ounces of blood were first drawn from the nape by cupping, it was then blistered, cold was applied to the scalp, and some purgative medicine was ordered; but three weeks afterwards he died, without having experienced any amendment. A large quantity of serum was deposited under the arachnoid, which was easily detached from the surface of the brain, except over the anterior and lateral parts of the left hemisphere; where, on tearing it off, some medullary matter in a completely softened state came away with it, and a quantity of cream-like fluid ran from the breach which had been thus made into the interior of the brain, where for three inches square it was softened into a state, which the Doctor was inclined to consider analogous to suppuration. In most parts the softened matter wore a yellowish color, and in some places the vascularity of the cerebral substance was much increased. In one small spot in the posterior lobe the same morbid tendency was betrayed.

The morbid anatomy of this case throws but little light upon the unexpected commencement and rapid course of the disease. When paralysis took place the man was engaged, in apparent health, by his ordinary occupations, and from the moment of its establishment until the close of life, there was no change of symptoms which could hold out any prospect of recovery.

"The question then is, what was the change which gave rise to the sudden aggravation of symptoms? It is certainly possible that at this moment a vessel gave way, and blood was effused into the substance of the brain, which was afterwards so far absorbed as to leave only a yellow appearance: this, however, is very improbable; for though we do not know the exact time necessary to absorb blood in the brain, still we know that frequently twenty-four days is quite insufficient for the purpose; and long after that time we find more obvious traces remaining than were found in this case, when if any effusion at all took place, it must have been extensive.—Another hypothesis is, that the sudden mischief might have occurred when the softening arrived at some particular part, possibly when it reached the corpus striatum. And another explanation which might account for the symptoms of local pressure on the brain would be, that the pressure was occasioned by an accumulation of the serum found beneath the arachnoid over the softened portion; or by the simple falling in of that portion forming the depression observed in its surface. It is not improbable that whenever a part of the substance of the brain is brought into a fluid state, or even an approach to that condition, it makes an undue pressure on the surrounding cerebral matter; and in this way may be produced many of the symptoms observed in affections of this kind." 173.

The last case is placed among those, which are intended to illustrate the effects of cerebral inflammation; but the peculiarity of its onset might have entitled it to rank among such instances of cerebral disease as depend on *Pressure*, and the morbid appearances, which it exhibited upon dissection, might appropriate to it a place among illustrations of *Softening of the brain*. The symptoms most generally present in this last affection differ but little from those which arise from pressure; but a softened condition of the brain may exist in many forms, originate in different states of disease, and be attended by a great variety of symptoms. In the first case, for example, which we shall extract, the symptoms came on as suddenly and the palsy was as complete as though blood had been effused in large quantity upon the surface of the brain. In the 82d case the attack was sudden but no palsy followed, and partial recovery afterwards took place. In the 81st and 82d cases, Dr. B. believes that softening of the brain arose from defective circulation; in the 65th case from inflammation; and in the 84th case from simple congestion; while instances are not unfrequent where the whole brain is generally softened in its texture, in consequence of great constitutional debility, or lengthened general disease.

The inducing causes being thus so various, our curative measures should be accordingly modified; but it is more than difficult in many instances to ascertain, either the precise morbid condition upon which the symptoms depend, or the precise exciting cause which gives rise to this condition.

(Case 81.) A man, aged 63, while sitting at breakfast was suddenly seized with a loss of consciousness, and upon coming to his senses ten minutes after, found that he had been attacked by complete hemiplegia of the right side, depriving him of all voluntary motion and sensation both in the upper and lower extremities. His mind was undisturbed, he could protrude his tongue, and his face was but slightly affected; but he swallowed with some impediment, and spoke with great indistinctness. For several years he had an open ulcer in the leg, which had healed up six months before his attack; since which his bowels were extremely irritable, and the diarrhœa, which this irritability occasioned, declined only on the sore becoming worse.

Nothing, which was done for him, had any effect upon his disease. He was occasionally delirious, his pulse was very intermittent, and becoming less sensible, whether from debility or increased pressure was uncertain, he gradually sunk. Beneath the arachnoid serum was effused in large quantity separating the convolutions from each other, and giving them a peculiarly rounded appearance by its lateral pressure. On the right side this appearance of the convolutions was most marked. The substance of the right hemisphere was natural, but on slicing the left at different depths the cortical substance appeared of a light fawn colour, very soft, and almost broken down into the general texture of the brain; while the medullary portion was curd-like, and appeared in little holes as if absorption had been rapidly at work. This disorganizing process seemed to begin near the cortex, and to proceed inwards. The ventricles were distended with water. A small portion of the centre of the left hemisphere of the cerebellum was also softened, and of a yellow fawn colour. The vessels at the base were ossified; but those on the pia mater and surface of the brain were not unnatural.

(Case 83d.) A young man, who had been from infancy subject to head-ache and giddiness, and who had had in China a paralytic attack, was placed under the Doctor's care in consequence of pain in the forehead, attended with dulness of sight and hearing. On laughing the mouth was drawn to the right side, and on attempting to whistle he was unable to close his lips on the left side. There was no fixed palsy of either side, but a general debility and loss of motive power. When he walked he staggered, and when he stood a few minutes on his legs he trembled. Tartar-emetic ointment was rubbed in upon his neck and shoulders, and the bowels were kept open with active aperients. A fortnight afterwards his head-ache had increased, he was often so giddy as to require to be led, and his countenance was inattentive and vacant. Leeches were repeatedly applied to his forehead, cold to his head, and a blister to his nape. Yet by the end of the following week he was comatose, had lost the power of his right hand, and constantly lay in whatever position he was placed. During the next fortnight he expired. The left hemisphere was covered with pia mater so highly vascular as to give to the whole a deep red colour; but there was no effusion. If the presence of a little serum, which lay beneath the arachnoid, be excepted, the right hemisphere appeared healthy. Two softened portions of brain—one in the anterior, the other in the posterior lobe—were discovered in the left hemisphere. The smaller portion was separated from the healthy brain by a distinct line, which seemed to be firmer than the general structure: the larger mass was less defined and involved both the cineritious and medullary textures. During the two days, which were occupied in making drawings of these parts, the different effects of the air upon the sound and diseased brain were so remarkable, that while the healthy part retained its firmness the softened portion became semifluid.

(Case 84th.) A man, 73 years of age, fell down in a fit of apoplexy on hearing that his partner had absconded, and reduced him from a state of affluence to penury. About ten months afterwards he had a second fit as severe as the first, after which he became unable to engage in any occupation, and about three quarters of a year afterwards he was attacked a third time, while endeavouring to get from his bed to his chair, for which he was liberally bled from the temporal artery. After this he became comatose, was occasionally delirious, lost much of the power of his right hand and lower extremities, which could not be extended without pain, and he died eight months afterwards. The inner surface of the dura mater was very soft, a quantity of serum lay between the pia mater and the arachnoid, the vessels of the pia mater were much injected, the medullary substance of the brain was brown-coloured, but firm. The ventricles were distended with serum, and lined with a few finely-injected vessels. At the base of the brain there was likewise much fluid. In each of the posterior lobes of the brain there was a spot of softening, in which both the cortical and medullary textures were involved. The centre of this softened mass was the least firm, but although its consistence grew harder as it extended towards the surface, there was no boundary line to separate the diseased from the healthy parts.

"In this case there is great reason to believe that no material vessel was at any time ruptured, but that the successive apoplectic fits depended rather on a state of congestion in the vessels generally, producing slight laceration of the fine branches, chiefly at the union of the cineritious and medullary matter, which probably never completely regained their natural condition after the first attack. At the same time no decided paralysis was the result of the first seizure; and though from time to time there was evidence of the return of a state of vascular fulness, marked by the temporary loss of memory and the confused state of mind, yet even his mind was generally capable of active employment for above a year, when the recurrence of a similar attack more completely destroyed his power of exertion both mental and bodily, still without inducing fixed paralysis. By the third attack, which took place about six weeks previous to his death, it is probable that the more complete disorganization of the injured portions of the brain was effected: decided paralysis followed, confined to particular parts and chiefly to the nerves of motion; and during the last three or four days he became completely insensible and comatose, from the pressure of the serum which was accumulating in the ventricles and beneath the arachnoid. It may fairly admit of a question, whether in this last attack the disease did not assume an inflammatory character. The quick and sharp pulse, the furred tongue, and the hot skin, all appeared to indicate the existence of inflammation; and the pulpy state of the arachnoid lining the dura mater, and the vascularity of the pia mater, seemed to point to the same condition, independently of the serous effusion and of the softened state of the portion of brain at the junction of the cineritious and medullary substances, which might possibly be the result of congestion." 188.

In the 85th and last case of "ramollissement" of the brain, an athletic soldier, aged 40, who wore upon his head several marks of preceding injury, came into Guy's Hospital on the 21st of January, labouring under general muscular and nervous debility. His left leg and arm were weak, his walk was unsteady, he passed his urine involuntarily, and he betrayed several proofs of a confused and injured mind. During the previous year he had complained of pain in the forehead, and he had fallen repeatedly while walking on the streets. On the 25th a seton was inserted in his nape, he had five grains of blue-pill at night, and a black draught in the morning; on the 30th ten leeches were applied to the temples; on the 6th of February one grain of the sulphate of zinc was given three time a day in addition to his other medicines; the sulphate of zinc was increased and continued until the 27th, when subcarbonate of iron was substituted in its room; quinine, valerian, nux vomica, tincture of Spanish flies, blistering the nape, and electricity were successively tried on to the 8th of July, without producing any serious alteration; and on the 23d he died in a state of such extreme helplessness, as to be unable to leave his bed or even swallow. The dura mater and arachnoid adhered in several parts, especially on the right side, so that they stripped off together; and over a considerable part the cineritious brain tore away with the membranes. There was decided serous effusion between all the convolutions, and the ventricles contained about an ounce of clear fluid. On the right side the corpus striatum was quite flattened and of a yellow color. It was soft to the touch, and when cut into two-thirds of it were broken down, and presented a filamentous watery brown appearance. Both kidneys unhealthy, but the right very much diseased; the superior half being of a yellow white color and containing two abscesses. His urine had been high-colored, and when exposed to heat became quite white and flaky.

It must easily be seen by a review of the above cases, that great difficulty will be encountered in constructing a diagnosis of this cerebral affection from the symptoms by which it is attended. Its actual presence in, perhaps, any instance can only be suspected; and even our suspicions depend upon the caprice of so many accidental occurrences, that to be well founded requires, probably, as much good fortune as sound experience. That softening of the brain, which occurs in cases of old age and general debility, which approaches slowly and only terminates in dissolution when the general frame is exhausted, the Doctor has not considered it necessary to illustrate by any examples. To those, who are conversant with morbid anatomy, no state of the brain is more frequently occurring, or better known; but, as it is merely one out of many effects of constitutional decay, the patient stands in general little stronger chance of recovery after his disease has been clearly ascertained, than previously.

"With regard to the treatment of cases in which we are induced to suspect that the softened disorganization is taking place, it will of course vary according to the cause: as long as we are obliged to infer excessive action we must deplete, and use counter-irritation; but looking to the general condition of those who are the subjects of such disorganization, we should be inclined to prohibit active depleting remedies, as likely to diminish the powers of the system. Nor can we suppose that the mercurial action would produce any good effect. On the contrary, whatever is calculated to disturb the more healthy and natural actions may be expected to do harm, and probably the careful avoidance of every thing which can over-excite either the body or the mind, with the employment of gentle tonic remedies, both as medicine and as diet, and even as occupation and amusement, will be most effectual in delaying the mischief, or in supporting the frame under its gradual decline. To what period life may be prolonged, when a considerable extent of the substance of the brain is diseased, we have not the means of asserting; but that large portions of the brain may be lost with apparent impunity has been proved in many instances; and in some of the foregoing cases there is reason to believe that the mischief had existed several months, attended however with great functional derangement. Whether healthy brain be ever regenerated is very doubtful; but as to the power of Nature of repairing in some degree the injuries of the brain there can be no doubt: and there is reason to believe that parts of the substance which have been lacerated by blows or by apoplexy, and thus rendered useless, are frequently absorbed; but how far the powers of the system are sufficient for such an operation, where, as in the cases of Kennedy or Murridge, spontaneous change has taken place, remains a matter of speculative opinion; at all events, one of the best objects we can propose in the treatment, is to give force and vigor to those natural actions by which such reparations are effected." 196.

The Doctor's *Second Section* is devoted to the different kinds, causes, and effects of *Pressure* upon the brain. Under this head are considered pressure from simple vascular turgescence;—pressure from the effusion of serum between the membranes, or into the ventricles;—pressure from extravasated blood; and pressure from various other varieties of accident. A plethoric, robust and well-fed man is suddenly seized after dinner with giddiness, indistinctness of vision and intense pain in the head, followed by loss of consciousness and voluntary power. He is bled from the arm or temples to 20 ounces, and in an hour or two giddiness, pain, and every other symptom disappear. In this instance there was evidently nothing more than intense cerebral congestion;—a larger quantity of blood in the vessels of the head than was compatible with the free performance of the sensorial functions. But this man, by persevering in his luxurious mode of life, is again attacked by a fit of confirmed apoplexy, from which he recovers slowly, and probably with the sacrifice of one or two palsied limbs. In this case we may suppose that the cerebral congestion had reached its most intense degree, and that the blood determined to the head, no longer to be contained within the bloated vessels of the brain, breaks through some of its natural channels, and not only overwhelms the nervous power for a time, but leaves serious and lasting vestiges of injury. Between these two states, which an ordinary eye may be able to distinguish, a third intervenes of more difficult diagnosis. A man instantly falls down, foams at the mouth, is violently convulsed, snores loudly, is quite insensible, and remains in this state for several hours; when, either after the use of the proper remedies, or without any kind of treatment, he gradually recovers; but feels languid and weak for months afterwards. Such cases are by no means rare, and it very generally happens that one such attack only prepares the way for others until the constitution is irreparably damaged. The features, by which such cases are distinguished, so correspond in many respects with those of epilepsy on the one hand, and of confirmed apoplexy from sanguineous effusion on the other, that the line of demarcation is not easily drawn, although such distinction is often of great practical importance.

(Case 87.) A gentleman, aged 60, of an apoplectic habit, was attacked at the billiard table with giddiness and indistinct vision; some time after which he fell senseless and convulsed upon the ground. An hour afterwards he was bled to 16 ounces, and took ten grains of calomel, which for a time relieved him; but early next morning a second attack occurred, and the bleeding was repeated. At this time he lay rather senseless, but apparently asleep. When roused he answered questions and again fell off to sleep. No limb was palsied, but the mouth appeared somewhat drawn to one side, and his pupils were contracted. Ice was applied to the head, and a purging injection was ordered; twelve ounces of blood were drawn from the arm during the night, and strong purgatives were given to stimulate the bowels, upon which the injection had no effect. During the 24th he had a third attack, for which he was cupped, leeched, and blistered.

On the 25th he was better, although perfectly lethargic when left to himself. On the 26th his improvement continued. On the 27th and 28th his mind was occasionally hysterical, but on the 29th he was so much better that the Doctor's attendance was no longer necessary. A slight paralytic affection of the left hand remained, his left eye was slightly affected, the left angle of his mouth was just perceptibly drawn up, and he occasionally complained of shooting pains in the back of his head. He afterwards became the subject of a fresh attack, which was overcome by similar means, but which left an imbecile and desponding state of mind that several months were spent in dissipating. In such cases—

"The most difficult point to be decided, generally, is the extent to which depletion should be carried. The various circumstances of the individual, but more particularly his habit of body, will afford the most certain guide; and in most cases, in proportion as the epileptic character prevails in the attack, will the propriety of large depletion become the more questionable. That very urgent vascular congestion exists during paroxysms of this mixed character, there is scarcely room to doubt; but in general, direct depletion is to be considered only a temporary means of relief, calculated to remove the present danger from fatal over-distention of vessels and from rupture; but if frequently or largely repeated will often increase the irritability and the tendency to relapse. When the present danger has been removed, establishing a regular action in the large intestines, and the employment of a tonic regimen and diet are most likely to prevent the repetition of the attacks." 202.

It is well known that upon inspecting the brain after death occasioned by narcotic poisons, its vessels are generally found in a very loaded state, and that such treatment as tends to prevent or remove cerebral congestion, has often the happiest effects in cases where poisoning has been attempted. How far such congestion is the immediate cause of death in any instance—whether it should be regarded as the effect of a less visible but more influential morbid state, or as directly contributing to depress and disturb the sensorial functions—is less certainly made out.

(Case 88.) A young woman, who had drunk a quantity of opium with the view of committing suicide, was brought into Guy's Hospital two hours after it had been swallowed. The stomach-pump and stimulants were tried in vain, and she expired in about seventeen hours after the accident occurred.

The vessels of the head were generally and unusually loaded—the surface of the anterior part of the left hemisphere contained a patch of ecchymosis equal to a crown-piece in size;—the substance of the brain was filled with bleeding points;—there was no water in the ventricles. Except two or three ecchymosed spots, which were ascribed to the tube of the pump, the stomach was healthy. The large intestines contained a few appearances of inflammatory action.

The next three cases, 89, 90, 91, are from the pen of Mr. Walne, and show in the most striking and satisfactory manner the influence of cold, as an application to the head, in cases where excessive doses of laudanum have been taken. This remedy, we believe, was first brought formally into notice by Mr. Wray and Dr. Copland, and the efficacy which these gentlemen's approval of it declared it to possess, should have given it a more general claim upon the adoption of the faculty than it appears as yet to enjoy. It has been known since the days of Hannibal, when the grapes of Italy were first tasted by his hungred Carthagenians, that pouring cold upon the body of a drunken man relieves him from his intoxication in a very short time; and the action and effects of brandy upon the brain, when taken in excess, do not appear to be extremely different from those of the more concentrated and less palatable narcotic poisons.

(Case 91.) A strong young man took upwards of an ounce of laudanum as he was retiring to rest, and in somewhat more than a quarter of an hour Mr. Walne saw him. The stomach-pump was introduced, and, after as much of the contents of the stomach was removed as possible an emetic was introduced but failed to operate. Cold water was now dashed over his head, and at each dash some signs of returning sensibility were elicited. In a little time he complained of being sick and vomited freely what had been thrown into his stomach. He occasionally relapsed into a state of stupor, but a fresh application of the dash was always sufficient to restore him again to sensibility. This treatment was continued between two and three hours, when he was rubbed dry and until his skin became quite warm. Headache was complained of for two or three days, but he perfectly recovered.

The pump in this instance strongly co-operated with the action of the cold, and in no instance should the water alone be trusted to. The pump may, however, be employed either after the narcotic has passed down into the intestines, or has brought the system under the full influence of its principle. In such cases emptying the stomach can do little good; but still it should not be neglected.

(Case 89.) A girl swallowed an ounce and a half of laudanum, and in twenty minutes she was in a state of complete insensibility. The pump was introduced, but very little fluid could be withdrawn; and that did not seem to contain any of the tincture. Water was abundantly injected, but the smell of opium could not be detected in it when afterwards discharged. Mugs-full of cold water were now poured upon her head, and occasionally upon her face. Symptoms of returning sensibility followed each application, but whenever the affusion was discontinued for a few minutes the patient relapsed into her previous state. A small quantity of ammonia was poured into her stomach, but she retched repeatedly and brought part of it up. At the end of about four hours the whole surface was cold, the pupils were contracted to the utmost, the action of the heart was feeble, and she was much less excited by the dash. The cold affusion was now discontinued, she was placed in a warm bed and rubbed dry, while hot bricks were applied to her feet. The friction roused the heart and restored warmth, but she continued insensible, and in less than two hours afterwards her jaw fell, the pulse became hardly perceptible, the breathing was marked by mucous rattle, and there was every symptom of approaching dissolution, when Mr. Walne determined on injecting some brandy into the rectum. Four ounces were accordingly introduced, but they were soon rejected; yet the pulse got up and the jaw returned to its natural position. Half an hour afterwards the same quantity was repeated with three quarters of a pint of gruel. This was retained and her pulse improved, she breathed freely, and she fell into a profound sleep, out of which she awoke so far recovered as to be no longer in a doubtful state.

This case is one of very considerable interest, for the favourable result of which Mr. Walne merits no small credit It shews what good can be accomplished by the patient and industrious use of judicious means, even *after* there is little ground for hope to rest upon. The excitability of this girl had been evidently exhausted by the continued application of the cold dash for four hours ; yet no one can for a moment doubt that, had not this application been made, she would not have survived one-third the time of its employment. The medicine had taken full possession of the system before medical assistance was obtained, and had evidently passed into the intestinal tube ; so that by the continued stimulus of the dash the vital sensibility was preserved unextinguished until the narcotic began to lose its influence upon the brain, when the restorative power of the brandy completed the cure by re-establishing the nervous energy. The cold was at first indispensable to counteract the effects of the narcotic ; and the brandy was as necessary to remove the debility, which had been occasioned by the conjoined operation of the narcotic and the cold.

Perhaps pulmonary affections are as frequently a cause of congestion within the head as any, which have yet been mentioned. In the 93d and 94th cases the bronchial membrane was seriously affected, attended by much dyspnœa and lividity of the countenance, and followed by incoherence and pain of head. The vessels of the brain were found turgid with blood, and the substance of this organ presented a mottled aspect, which chiefly depended on its increased vascularity. The 95th, 96th, and 97th cases indicate the operation of pulmonary emphysema upon the cerebral circulation. The principal symptoms were dyspnœa, great difficulty in walking to any distance, respiration generally inaudible by the stethoscope, with a clear and loud thoracic sound on percussion. The 96th case is especially interesting on account of its well-marked and characteristic nature ; but space will not permit us to do more than thus refer it to the reader's notice. The 98th and 99th cases are from the practice of Mr. Walne, and are very distinct instances of the effects of pulmonary obstruction, accompanied by hooping-cough, upon the circulation within the head. In the 99th case, had the patient died of pure apoplexy, the brain could not have displayed more striking vestiges of that disease. The lungs were condensed, hepatized and infiltrated. The conditions of the lungs, which seem most calculated to induce the cerebral plethora, are—

"Condensation from the pressure of effused fluid ;—changes in the bronchial membrane from chronic inflammation ;—extensive emphysema of the lungs, whether the consequence of original weakness in the structure of those organs, or from violent exertion, or from chronic thickening of the bronchial tubes ;—and sanguineous congestion generally dependent upon some obstruction to the free passage of blood through the heart ;—and occasionally the changes consequent on Phthisis and Pneumonia. Many of the most distressing symptoms of bronchitis,—the intense headache, the wandering delirium, and the lethargic coma,—are undoubtedly dependent upon the state of the circulation through the head. (See 1st volume of these Reports, p. 27 to p. 34.) It is however not quite evident what part the simple mechanical congestion and what part the chemical condition of the blood takes in this morbid train of symptoms : there can be little doubt that both these causes exert a hurtful influence ; for if any organ of the body is calculated to feel more injuriously than another the imperfect quality as well as the disproportionate quantity of the blood with which it is nourished, it is probably the brain ; and I shall hereafter, when speaking on the subject of Jaundice, take the opportunity of referring to that disease as another instance in which the imperfect state of the blood is found to influence the appearance of the brain most obviously, and, as far as we can judge from the symptoms of mental and bodily depression, to influence the functions of that organ. In the case of jaundice, however, the evidence of pressure and mechanical accumulation is much less distinct, than of functional derangement depending upon the altered condition of the blood." 221.

But here we must lay down our pen, and bid the Doctor adieu for the present number. We have devoted no mean share of our space to the present article, and we have labored as much as in us lay to collect and condense, into the smallest reasonable compass, the important facts and cases which came before us; yet two hundred pages only have been examined, leaving more than twice the number for some future opportunity; and we have habitually refrained from interparagraphing our analysis either with much comment or critique, that the original might enjoy the length and breadth of our review, undiminished and unencumbered. We are indeed sorry that the Doctor has not varied his cases with more general remarks and individual deductions; with more practical inferences and pathological principles. It may be wise in a young and inexperienced observer to deal more in premises than conclusions, but a writer of Dr. Bright's calibre is only expected to lay down premises for the purpose of drawing inferences from them.* It is, no doubt, a service of essential moment diligently to collect and faithfully to narrate the interesting cases of disease which come within our notice; but it is still a much more essential service to digest these cases into practical lessons, and to draw from them the real theory and treatment of disease. The mere journalist may achieve the one work;—none but the enlightened and experienced physician can perform the other; and certain we are that no physician is better qualified to philosophize upon his own cases and extract from them all the therapeutic value which they contain, than the author of the work before us. The second volume is much less objectionable than the first in this respect; and, therefore, this passing observation is to be considered as principally retrospective. We shall return to the work in our next Number.

* Modesty in *such* a pen cannot be esteemed a virtue.

